# Supplementary material for: A C21-steroidal derivative suppresses T-cell lymphoma in mice by inhibiting SIRT3 via SAP18-SIN3
Source: Commun Biol. 2020 Dec 3;3:732. doi: 10.1038/s42003-020-01458-3 (PMC7713351; doi:10.1038/s42003-020-01458-3)
Supplement: Supplementary file 1 — Reporting Summary [file 42003_2020_1458_MOESM1_ESM.pdf]

nature research

Corresponding author(s): Yaacov Ben-David, XiaoJiang Hao, LieJun Huang

Last updated by author(s): Sep 30, 2020

Reporting Summary

Nature Research wishes to improve the reproducibility of the work that we publish. This form provides structure for consistency and transparency in reporting. For further information on Nature Research policies, see our Editorial Policies and the Editorial Policy Checklist.

Statistics

For all statistical analyses, confirm that the following items are present in the figure legend, table legend, main text, or Methods section.

n/a	Confirmed	
☐	☒	The exact sample size (n) for each experimental group/condition, given as a discrete number and unit of measurement
☐	☒	A statement on whether measurements were taken from distinct samples or whether the same sample was measured repeatedly
☐	☒	The statistical test(s) used AND whether they are one- or two-sided<br>*Only common tests should be described solely by name; describe more complex techniques in the Methods section.*
☒	☐	A description of all covariates tested
☒	☐	A description of any assumptions or corrections, such as tests of normality and adjustment for multiple comparisons
☐	☒	A full description of the statistical parameters including central tendency (e.g. means) or other basic estimates (e.g. regression coefficient) AND variation (e.g. standard deviation) or associated estimates of uncertainty (e.g. confidence intervals)
☐	☒	For null hypothesis testing, the test statistic (e.g. F, t, r) with confidence intervals, effect sizes, degrees of freedom and P value noted<br>*Give P values as exact values whenever suitable.*
☒	☐	For Bayesian analysis, information on the choice of priors and Markov chain Monte Carlo settings
☒	☐	For hierarchical and complex designs, identification of the appropriate level for tests and full reporting of outcomes
☒	☐	Estimates of effect sizes (e.g. Cohen's d, Pearson's r), indicating how they were calculated

Our web collection on statistics for biologists contains articles on many of the points above.

Software and code

Policy information about availability of computer code

Data collection	Explained in supplementary note.
Data analysis	Graphs and statistical analyses were prepared using Graphpad prism (version 8). qPCR data were analyzed using StepOnePlus™ software (Version 2.3). Western blot (RD value) data were analyzed using ImageJ software. Flow cytometry samples were acquired using BD Accuri C6 software and the data were analyzed using FCS Express™ and FlowJo™.

For manuscripts utilizing custom algorithms or software that are central to the research but not yet described in published literature, software must be made available to editors and reviewers. We strongly encourage code deposition in a community repository (e.g. GitHub). See the Nature Research guidelines for submitting code & software for further information.

Data

Policy information about availability of data

All manuscripts must include a data availability statement. This statement should provide the following information, where applicable:

- Accession codes, unique identifiers, or web links for publicly available datasets
- A list of figures that have associated raw data
- A description of any restrictions on data availability

The authors declare that all relevant data supporting the findings of this study are available within the manuscript and its Supplementary Information.

Field-specific reporting

Please select the one below that is the best fit for your research. If you are not sure, read the appropriate sections before making your selection.

- [x] Life sciences
- [ ] Behavioural & social sciences
- [ ] Ecological, evolutionary & environmental sciences

For a reference copy of the document with all sections, see nature.com/documents/nr-reporting-summary-flat.pdf

Life sciences study design

All studies must disclose on these points even when the disclosure is negative.

Sample size	Sample sizes were determined based on previously published work and the work standardized by our research group.
Data exclusions	No data were excluded from the analysis.
Replication	In vitro: Three different individual experiments were performed with each experiments containing 3 replicates. In vivo: Experimental studies were performed 3 times.
Randomization	No specific method of randomization was used. However, for drug treatment experiments, after erythroleukemia induced by intraperitoneally (IV) with F-MuLV on newborn BALB/c mice and and C57BL/6 mice (2 months old), cages were randomly allocated to each group after balancing of sex.
Blinding	The investigators were not blinded to group allocation during data collection and analysis.

Reporting for specific materials, systems and methods

We require information from authors about some types of materials, experimental systems and methods used in many studies. Here, indicate whether each material, system or method listed is relevant to your study. If you are not sure if a list item applies to your research, read the appropriate section before selecting a response.

Materials & experimental systems

n/a	Involved in the study	
☐	☒	Antibodies
☐	☒	Eukaryotic cell lines
☒	☐	Palaeontology and archaeology
☐	☒	Animals and other organisms
☒	☐	Human research participants
☒	☐	Clinical data
☒	☐	Dual use research of concern

Methods

n/a	Involved in the study	
☒	☐	ChIP-seq
☐	☒	Flow cytometry
☒	☐	MRI-based neuroimaging

Antibodies

Antibodies used	Polyclonal rabbit antibodies for FLI1 (ab133485), BCL2 (ab), SIRT3 (ab217319), SIN3A (ab129087), cMYC (ab32072)obtained from Abcam, Cambridge, UK; ERK (#9102), phospho-ERK (#9101), AKT (#9272), phospho-AKT (#13038), GAPDH (#2118) antibodies obtained from Cell Signalling Technology (CST), Danvers, MA01923; SAP18 (13841-1-AP) from Proteintech, USA; goat-anti-mouse and goat anti-rabbit HRP-conjugated antibodies obtained from Promega, Madison, Wisconsin, USA.
Validation	Data provided in the manuscript.

Eukaryotic cell lines

Policy information about cell lines

Cell line source(s)	293T (ATCC® CRL-3216™)<br>EL4 (ATCC® TIB-39™)<br>CEM-C7H2 (Provided by Dr. Eitan Yefenof, Hebrew University, Israel)<br>MOLT-4 (ATCC® CRL-1582™)<br>BW5147 (ATCC® TIB-48™)<br>Raji (ATCC® CCL-86™)<br>OCI-Ly10 (RRID:CVCL_8795)<br>TK6 (ATCC® CRL-8015™)<br>BJAB (ThermoFisher Scientific, Cat. no. V04450)

	HEL (ATCC® TIB-180™)<br>K562 (ATCC® CCL243™)<br>DP17-17 (Generated by us)<br>CB7 (Generated by us)<br>CB3 (Generated by us)<br>RPMI 8226 (ATCC® CCL-155™)<br>WM9 (Rockland; WM9-01-0001)<br>WM239 (Rockland; WM239A-01-0001)<br>MCF7 (ATCC® HTB-22™)<br>MDA-MB-231 (ATCC® HTB-26™)<br>4T1 (ATCC® CRL-2539™)<br>FE1.2 (Generated by us)<br>FE1.3 (Generated by us)<br>PC-3 (ATCC® CRL-1435™)<br>BPH (Merckmillipore, Cat. no. SCC256)
Authentication	After obtained the cell lines, examined the cells morphology under microscope and tested mycoplasma free.
Mycoplasma contamination	All cell lines tested (MycoAlert™ PLUS Mycoplasma Detection Kit) negative for mycoplasma contamination.
Commonly misidentified lines (See ICLAC register)	We didn't use any commonly misidentified cell lines in this study.

Animals and other organisms

Policy information about studies involving animals; ARRIVE guidelines recommended for reporting animal research

Laboratory animals	Erythroleukemia induced by intraperitoneally (IV) with F-MuLV on newborn BALB/c mice and C57BL/6 mice (2 months old); Laboratory-bred strain; Male & Female.
Wild animals	Study did not involve wild animals.
Field-collected samples	Study did not involve samples collected from the field.
Ethics oversight	The animal protocol was reviewed and approved by the Guizhou Medical University Animal Care Committee under the guidelines of the China Council of Animal Care (Approval ID #1900373).

Note that full information on the approval of the study protocol must also be provided in the manuscript.

Flow Cytometry

Plots

Confirm that:

- [x] The axis labels state the marker and fluorochrome used (e.g. CD4-FITC).
- [x] The axis scales are clearly visible. Include numbers along axes only for bottom left plot of group (a 'group' is an analysis of identical markers).
- [x] All plots are contour plots with outliers or pseudocolor plots.
- [x] A numerical value for number of cells or percentage (with statistics) is provided.

Methodology

Sample preparation	For apoptosis detection, cells (1 × 106 cells) were washed twice with cold PBS and resuspended in 1ml of 1X binding buffer. Then transferred 100 µl of the solution (1 × 105 cells) to culture tubes (2 ml) and stained by FITC Annexin V (5 µl)/PI (5 µl) apoptosis detection kit. The cells gently mixed by vortex, incubated for 15 min at RT (25°C) in the dark, then 1X Binding Buffer (400 µl) was added into each tube and analyzed by flow cytometry within 1 h. For cell cycle analysis, cells were fixed by cold 75% ethanol overnight at -20°C, washed with cold PBS, stained in PI for 40 min at 25°C, then analyzed.
Instrument	BD Biosciences, Franklin Lakes, NJ and ACEA NovoCyte 2040R, USA
Software	BD Accuri C6 software and the data were analyzed using FCS Express™ and FlowJo™<br>NovoExpress software
Cell population abundance	Post-sort cell populations were generally >95% pure as per flow cytometer. Out of 1 × 106 cells, 1 × 105 cells were stained for analysis.
Gating strategy	See supplementary information.

- [x] Tick this box to confirm that a figure exemplifying the gating strategy is provided in the Supplementary Information.
