# Supplementary material for: A C21-steroidal derivative suppresses T-cell lymphoma in mice by inhibiting SIRT3 via SAP18-SIN3
Source: Commun Biol. 2020 Dec 3;3:732. doi: 10.1038/s42003-020-01458-3 (PMC7713351; doi:10.1038/s42003-020-01458-3)
Supplement: Supplementary file 2 — Supplementary Information [file 42003_2020_1458_MOESM2_ESM.pdf]

Supplementary information

A C21-steroidal derivative suppresses T-cell lymphoma in mice by inhibiting SIRT3 via SAP18-SIN3

Babu Gajendran[1,2], Krishnapriya Madhu Varier[3], Wuling Liu[1,2], Chunlin Wang[1,2], Klarke M Sample[4], Eldad Zacksenhaus[5,6], Cui Juiwei[7], LieJun Huang[1,2*], XiaoJiang Hao[1,2*], Yaacov Ben-David[1,2*]

Supplementary Figures

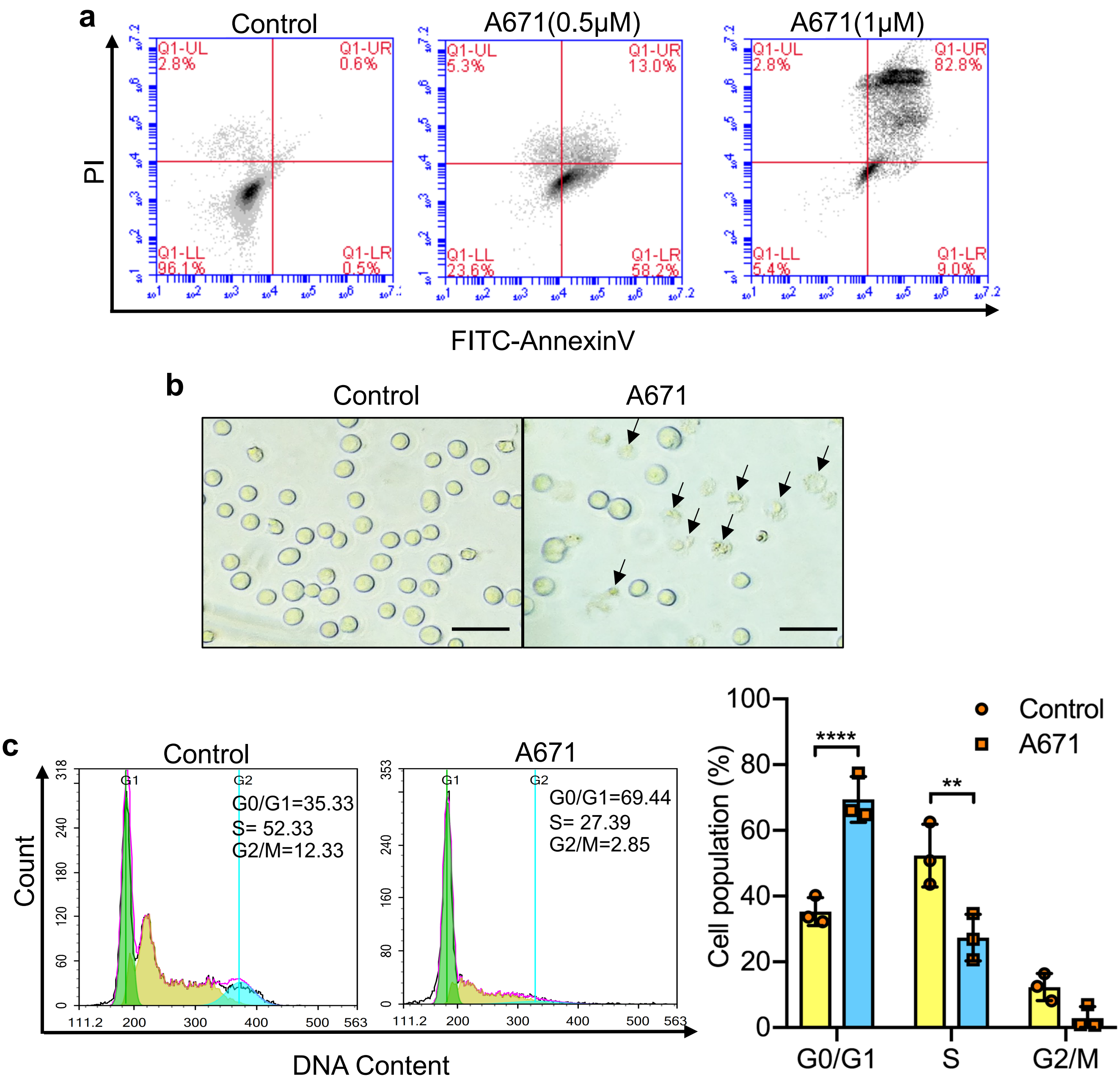

Supplementary Fig. 1 | A671 induces apoptosis in HEL cells in a dose-dependent manner. a, HEL cells were incubated with the indicated concentration of A671 and the apoptosis was determined by flowcytometry using Propidium Iodide and AnnexinV-FITC staining. **b,** The images of apoptotic effect of A671 on HEL cells. Arrows show apoptotic cells (Magnification ×100). **c,** Cell cycle profiles of the HEL drug-treated and control cells.

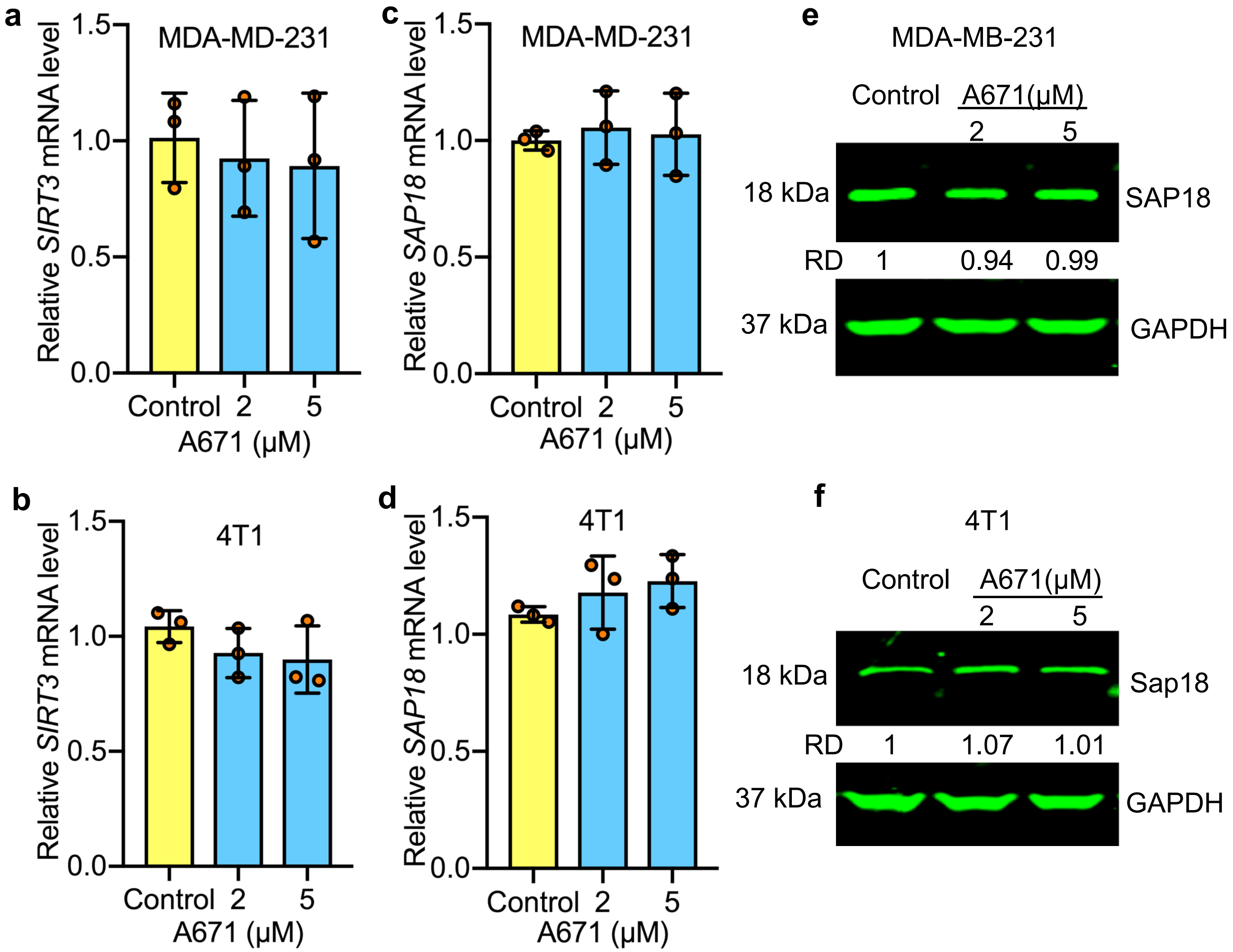

Supplementary Fig. 2 | Expression of SAP18 and SIRT3 in breast cancer cell lines after treatment with A671. a-d, Q-RT-PCR analysis of SAP18 and SIRT3 in MDA-MB-231 and 4T1 cells after treatment with A671 for 24 h. **e,f,** Western blot of extract from the indicated cells treated A671.

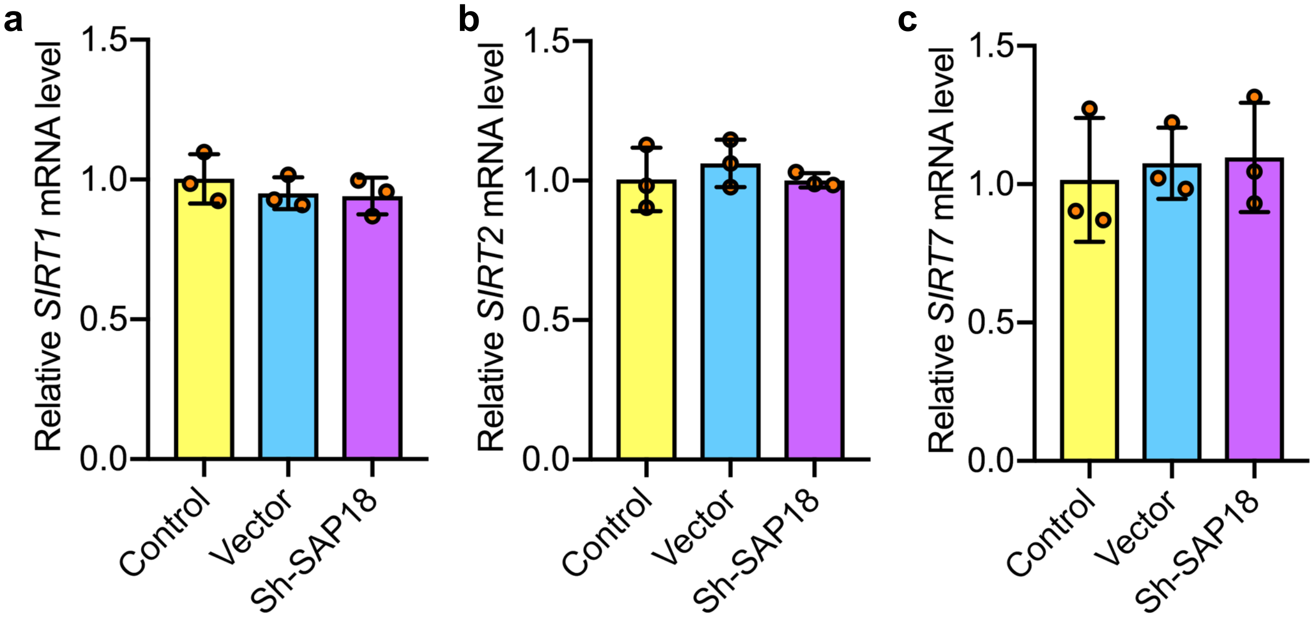

Supplementary Fig. 3 | Expression of the *SIRT* genes in SAP18 depleted HEL cells. HEL cells alone or after transfection with Sh-SAP18 or scrambled vector subjected to Q-RT-PCR analysis for the expression of SIRT1 **(a)**, SIRT2 **(b)** and SIRT7 **(c)**.

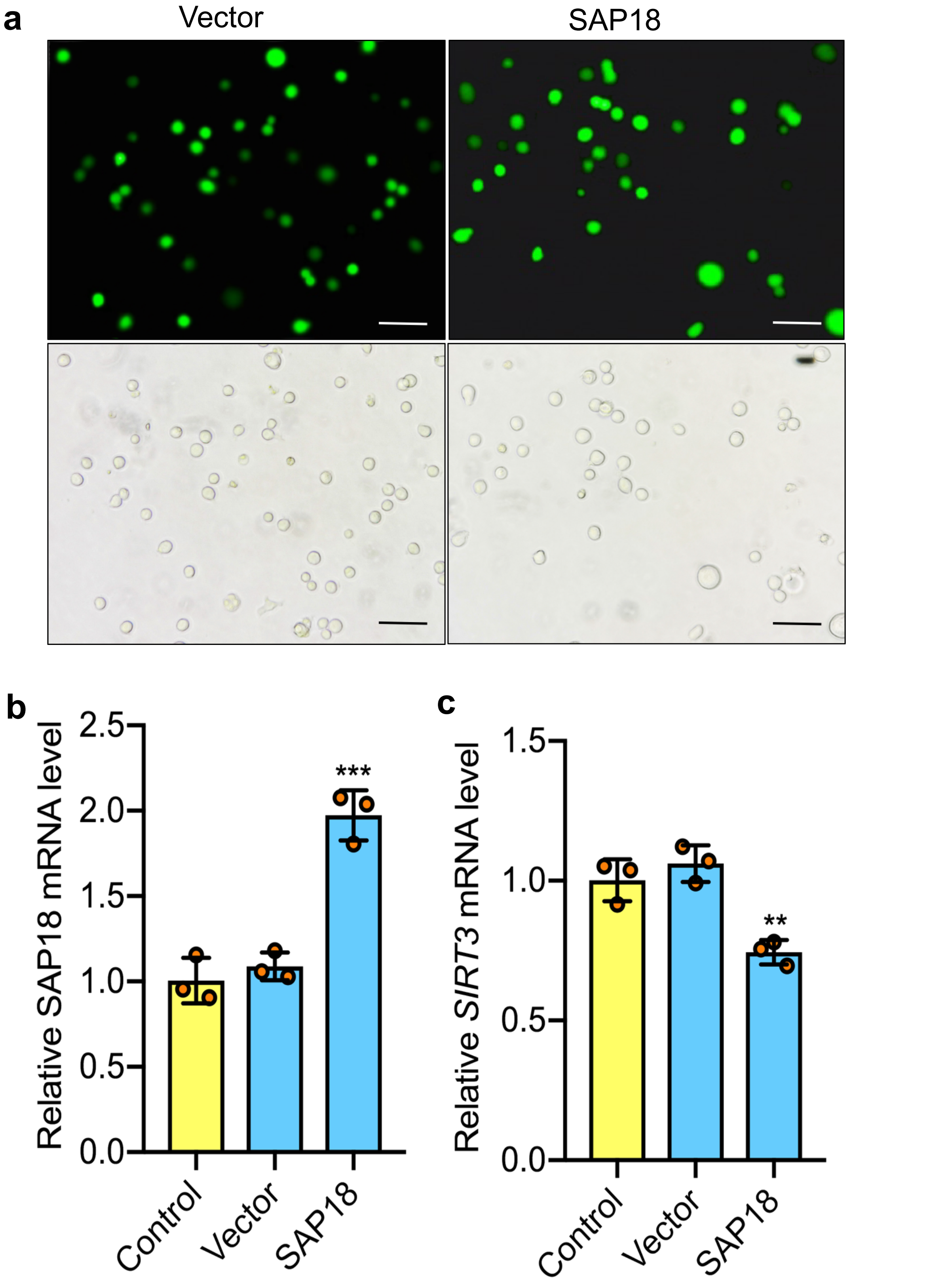

Supplementary Fig. 4 | Overexpression of SAP18 in HEL cells suppresses SIRT3 transcription. a, Images of HEL cells transfected with SAP18 (pCMV3-SAP18-GFPSpark®-KpnI-XbaI) and vector (pCMV3-N-GFPSpark®-KpnI-XbaI) plasmids illustrated for GFP (top) and microscopic view (bottom). Magnification ×100. **b,c,** HEL cells transfected with vector, SAP18 and none used to determine the expression of *SAP18* **(b)** and *SIRT3* **(c)** using Q-RT-PCR. P = <0.01 (**), P = <0.001 (***) by two-tailed student t-test.

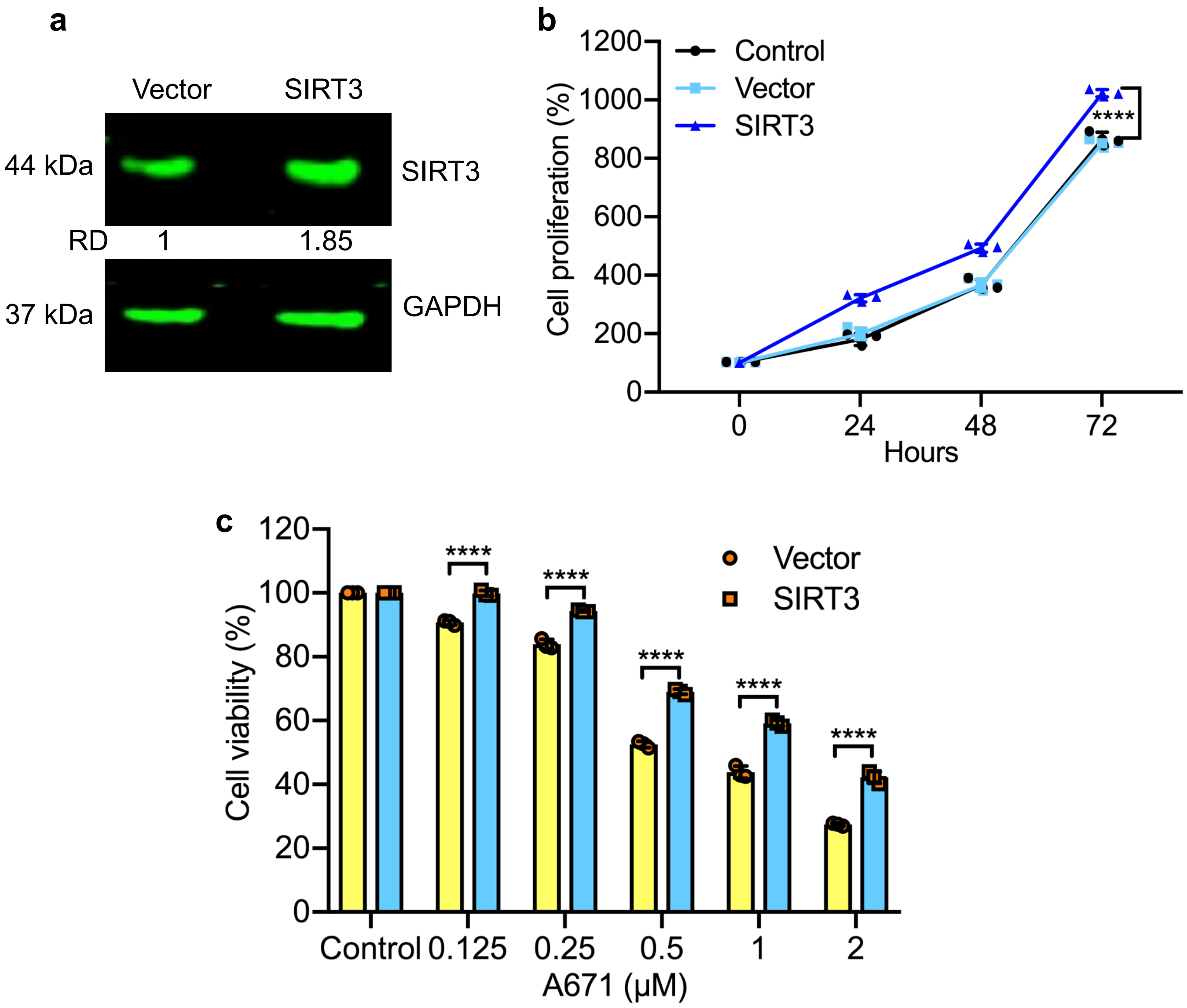

Supplementary Fig. 5 | Overexpression of SIRT3 in cells reduces cell toxicity by A671. a, Western blot of HEL cells transfected with SIRT3 (pcDNA3.1hSIRT3_H248Y_HA) or vector (pcDNA3.1) expression plasmids. **b,** Cell proliferation significantly increased in SIRT3 overexpression HEL cells versus vector-transfected cells. **c,** SIRT3-overexpressing HEL cells survive significantly at a higher rate than vector alone transfected cells after treatment with the indicated concentration of A671 for 24 h. P = < 0.0001 (****) by two-tailed student t-test.

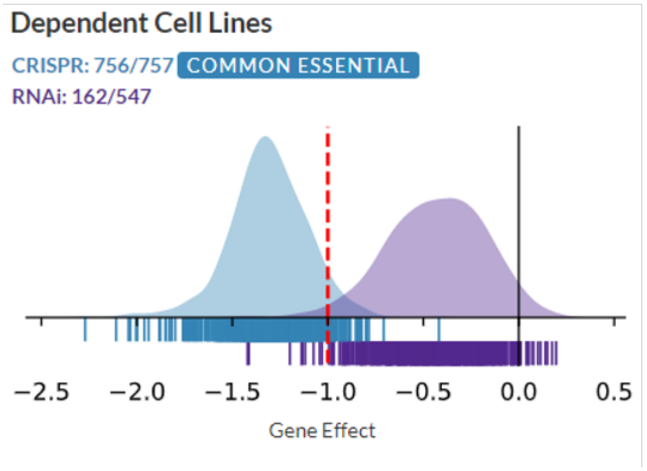

https://depmap.org/portal/gene/SAP18?tab=overview

SAP18

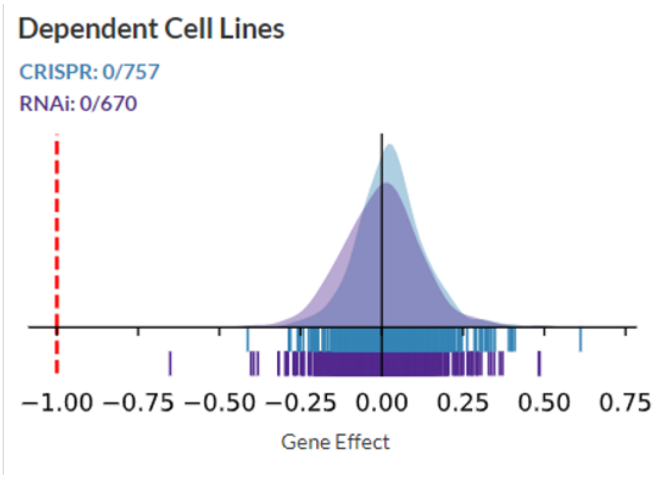

https://depmap.org/portal/gene/SIRT3?tab=overview

SIRT3

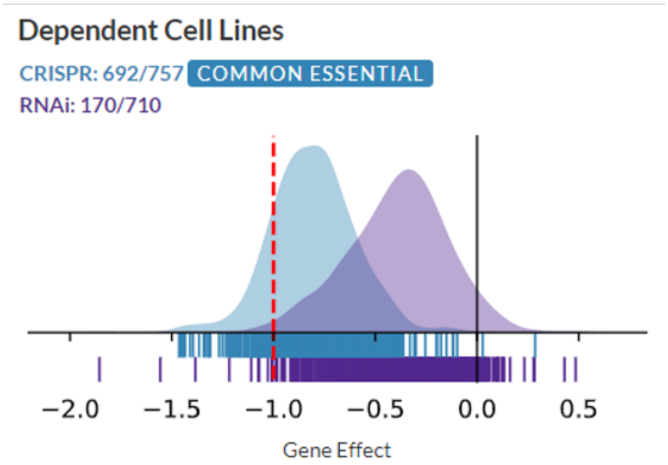

https://depmap.org/portal/gene/SIN3A?tab=overview

SIN3

Supplementary Fig. 6 l Survival rate of SAP18, SIRT3 and SIN3 in cell lines. The DepMap CRISPR data for SIRT3/SAP18/SIN3 across the hundreds of cancer cell lines.

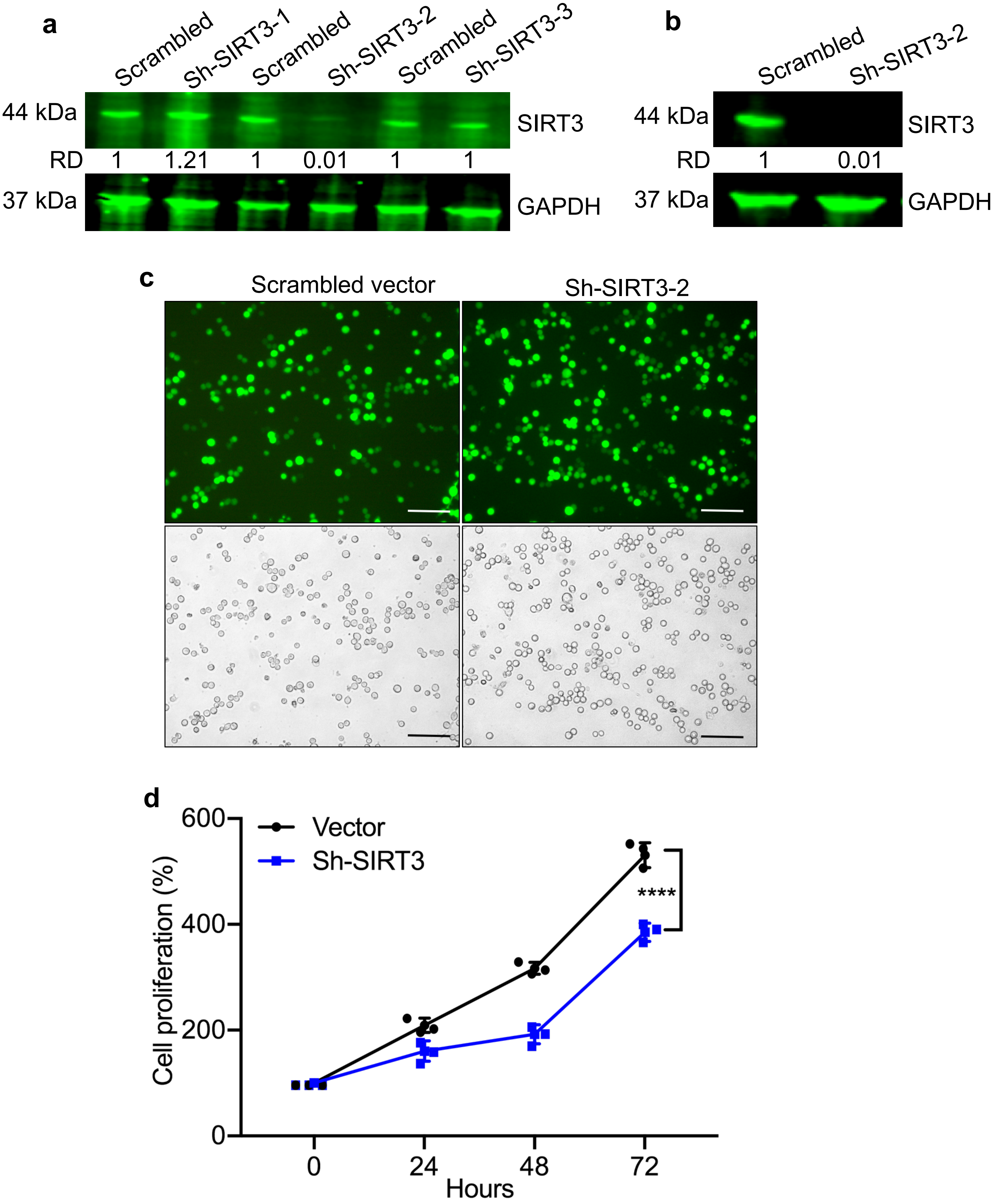

Supplemental Fig. 7 I Knockdown of SIRT3 in HEL cells reduced cell proliferation. a, Expression of SIRT3 after transfection with three shRNA for SIRT3 (sh-SIRT3-1, sh-SIRT3-2, sh-SIRT3-3). **b,** Expression of SIRT3 in shSIRT3-2 versus scrambled transfected cells. **c,** Microscopic image of GFP-positive sh-SIRT-2 and control transfected cells. Magnification ×40. **d,** Proliferation rate of sh-SIRT-2 and vector control.

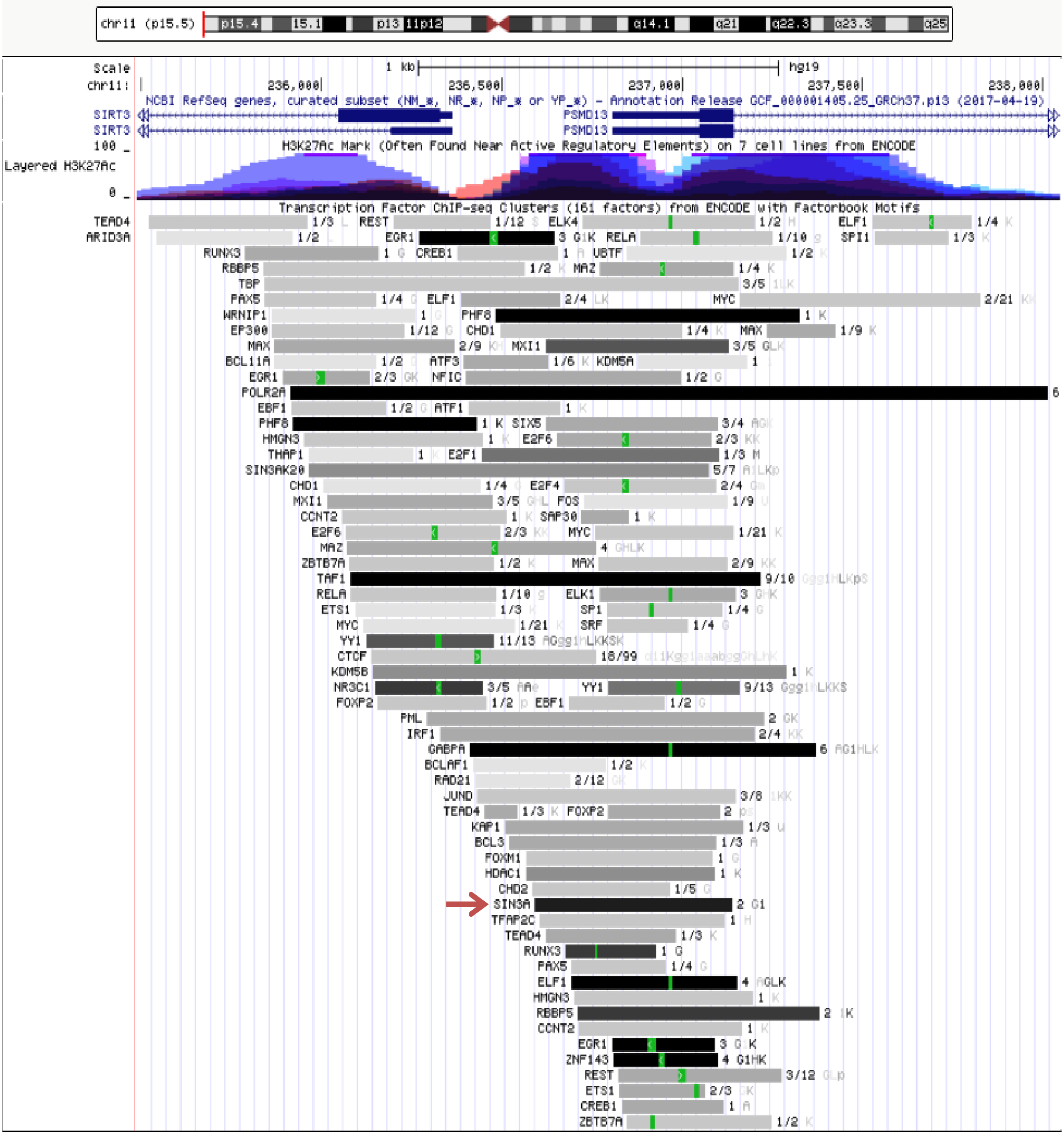

Supplementary Fig. 8 | SIN3A transcription factor binding site within the SIRT3 promoter. Graphical mapping of the ENCODE data to the GRCh37 (hg19) genome from the UCSC genome browser. A strong SIN3A transcription factor binding site is present within the SIRT3 promoter (as indicated by the red arrow, transcription factors with strong interactions are in black; weak interactions are in grey). The SIN3A binding site appears to be acting on SIRT3 (on the antisense strand) and not the neighboring PSMD13 (on the sense strand), since the signal is upstream of SIRT3 5' UTR and within the H3K27Ac mark, which indicates the boundaries of the SIRT3 promoter region.

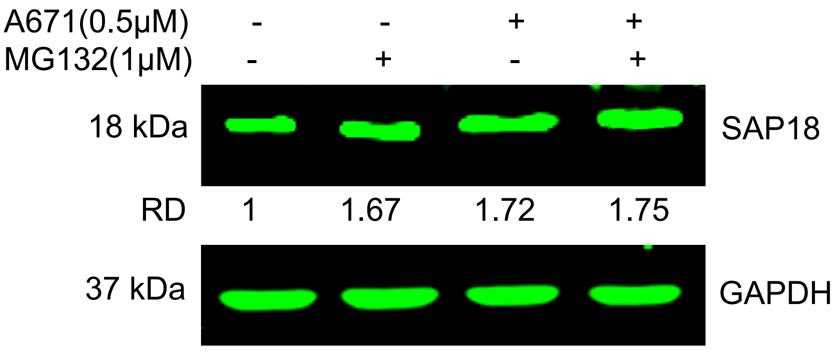

Supplementary Fig. 9 | Western blot of SAP18 expression after treatment with proteasome inhibitor MG132. HEL cells were treated for 12 h with A671 with or without additional treatment with MG132. GAPDH used as loading control.

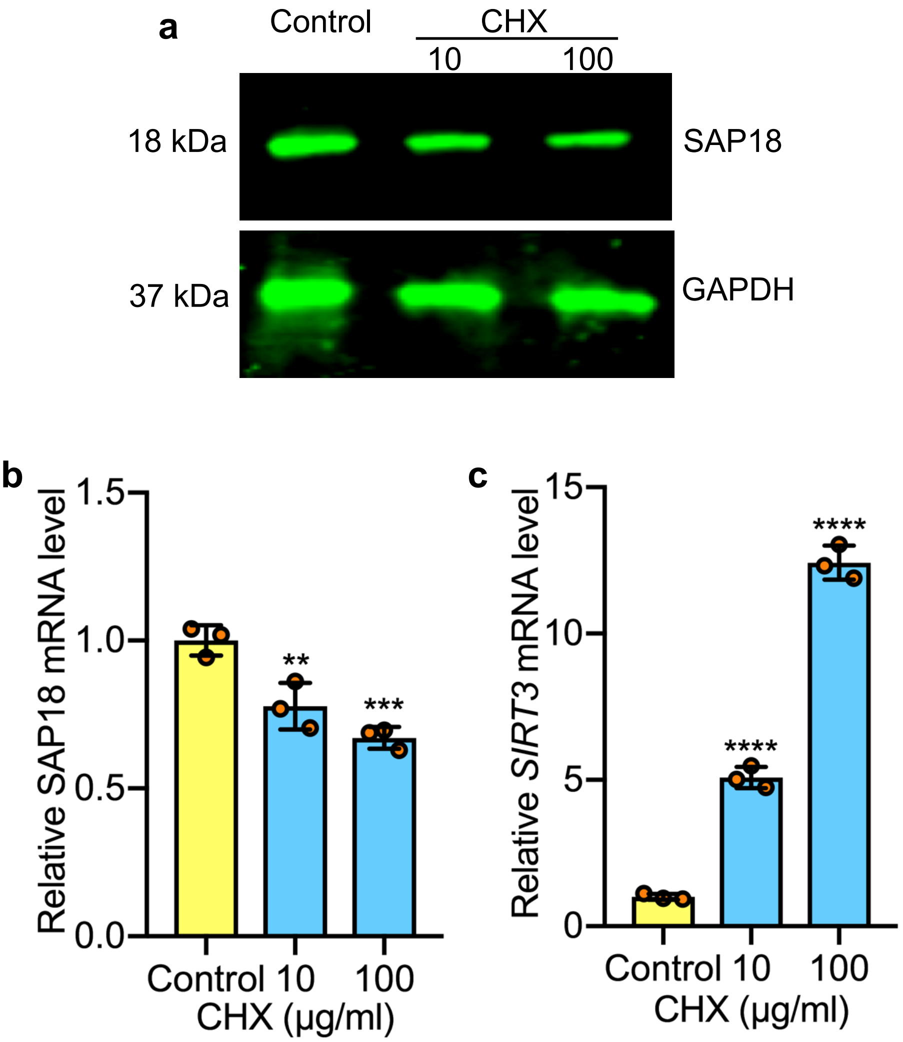

Supplementary Fig. 10 | SAP18 regulates its own transcription. a, Western blot of HEL cells treated for 24 h with the indicated concentration of Cycloheximide (CHX). **b,c,** Expression of *SAP18* **(a)** and *SIRT3* **(b)** after treatment of HEL cells for 24 h with the indicated concentration of CHX, as determined by Q-RT-PCR. P = <0.01 (**), P = <0.001 (***), P = < 0.0001 (****) by two-tailed student t-test.

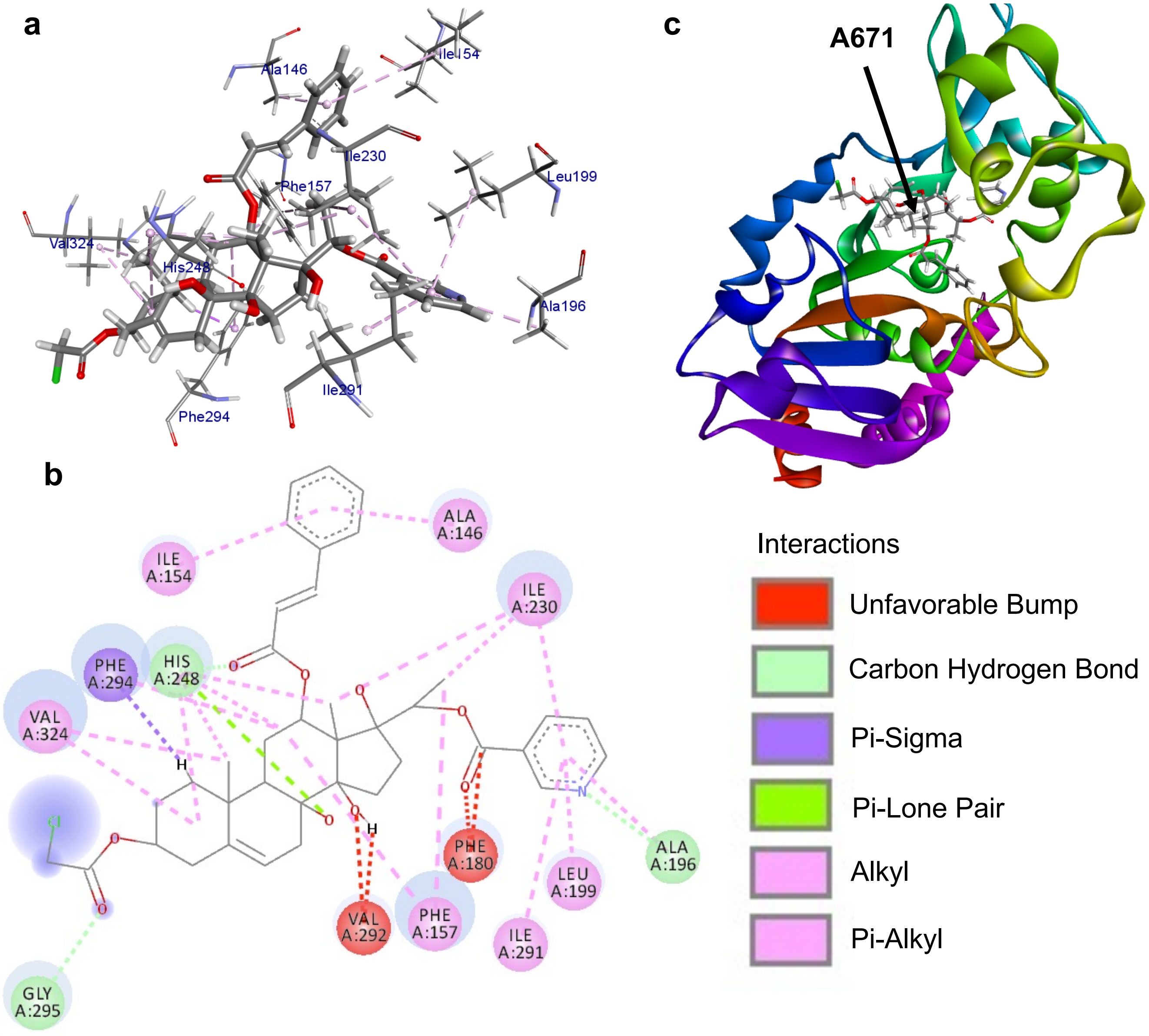

Supplementary Fig. 11 | The binding model of A671 and SIRT3. a,b, Two-dimensional interaction of A671 with various amino-acid within Sirt3. **c,** A 3D sketch of A671 interactions with Sirt3.

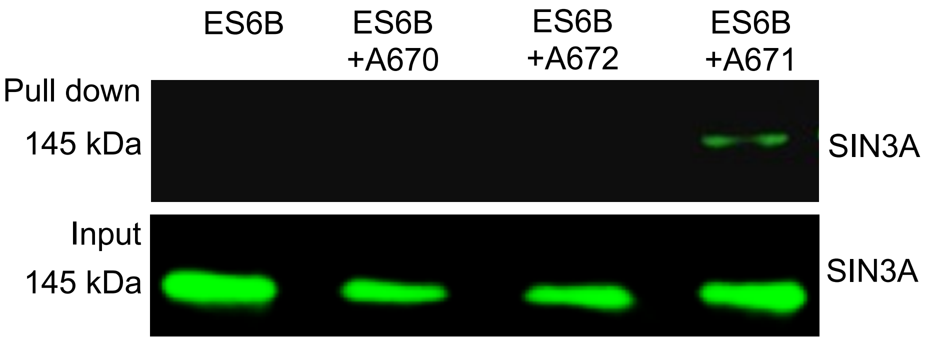

Supplementary Fig. 12 | Co-precipitation of SIN3A in the A671 pull-down experiment. In Pull down experiment, SIN3A protein found in SIN3/SAP18 complex precipitated with A671, while its presence was absent in A670 and A672 pull down.

Supplementary Tables

Supplementary Table 1 | Primers of real time PCR

Gene	Sequences		Species
	Forward	Reverse	
SIRT1	GGACATGCCAGAGTCCAAGT	GCTGGTGGAACAATTCCTGT	Human
	CGGCTACCGAGGTCCATATAC	CCGCAAGGCGAGCATAGATA	Mouse
SIRT2	CCGGCCTCTATGACAACCTA	GGAGTAGCCCCTTGTCCTTC	Human
	AGCCAACCATCTGCCACTAC	CCAGCCCATCGTGTATTCTT	Mouse
SIRT3	TCCACATCCCAATTCTGACA	TGGCCCTGACTGTAAACACA	Human
	GGGAGTGTTACAGGTGGGAG	AAGGGCTTGGGGTTGTGAAA	Mouse
SIRT5	AGTGGTGTTCCGACCTTCAG	CATCGATGTTCTGGGTGATG	Human
	CTCACGTGGTGTGGTTTGGA	ACAGGGCGGTTAAGAAGTCC	Mouse
SIRT7	CTCACCCACATGAGCATCAC	GGAACGCAGGAGGTACAGAC	Human
	GCATCACCCGTTTGCATGAG	GGCAGTACGCTCAGTCACAT	Mouse
SAP18	TAGGTAGTGGCGTGCATCTG	TTCTCATTTGTCAGGCACCA	Human
	AAGGGGTGTGTGGAAGTCAG	GACAATCGGAAAAACCCAGA	Mouse

Supplementary Table 2 | The sequence of SAP18 shRNA

Gene	Sequence	Species
SAP18 shRNA1	5’GATAGGAGATTACTTGGACATTTCAAGAGAATGTCCAAGTAATCTCCTATCTTTTTT-3’	Human
SAP18 shRNA2	5’CCTTGAAAGAACTGACAAGCTTTCAAGAGAAGCTTGTCAGTTCTTTCAAGCTTTTTT-3’	Human
SAP18 shRNA3	5’CCACCACCGAATGGACGAGTTTTCAAGAGAAACTCGTCCATTCGGTGGTGGTTTTTT-3’	Human
SIRT3 shRNA1	5’GCGGCTCTACACGCAGAACATTTCAAGAGAATGTTCTGCGTGTAGAGCCGCTTTTTT’3	Human
SIRT3 shRNA2	5’GTGGGTGCTTCAAGTGTTGTTTCAAGAGAACAACACTTGAAGCACCCACTTTTTT’3	Human
SIRT3 shRNA3	5’GCTCGGCATCTGTTGGTTACATTCAAGAGATGTAACCAACAGATGCCGAGCTTTTTT’3	Human

Supplementary Table 3 | Details of overexpression plasmids of SIRT3 and SAP18

Gene Name	Gene ID	Catalog	Species
SIRT3	NM_012239	24492	Human
SAP18	BC030836	HG14926-ANG	Human

Data for uncropped western blots

Figure 2a

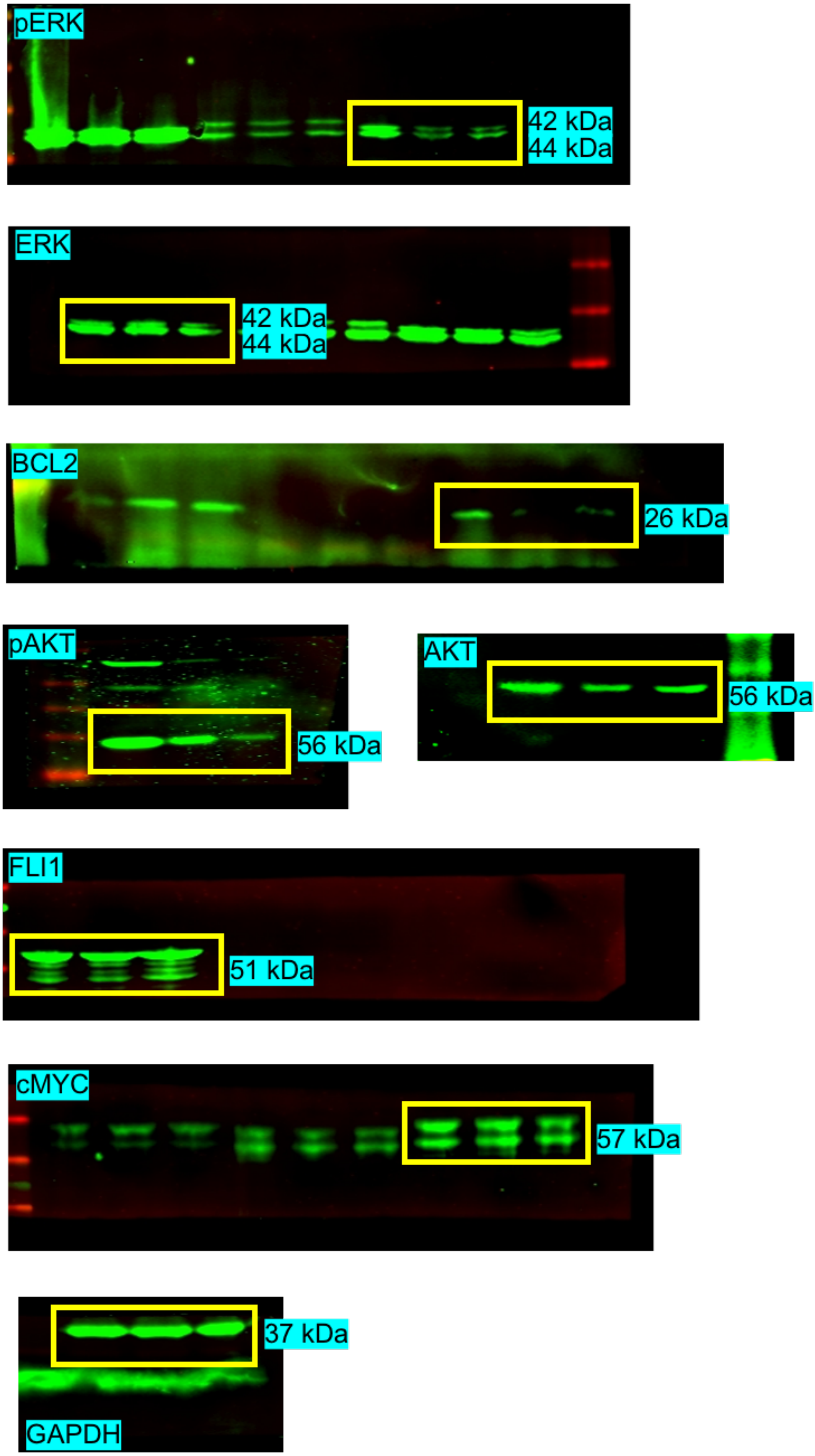

Figure 3I

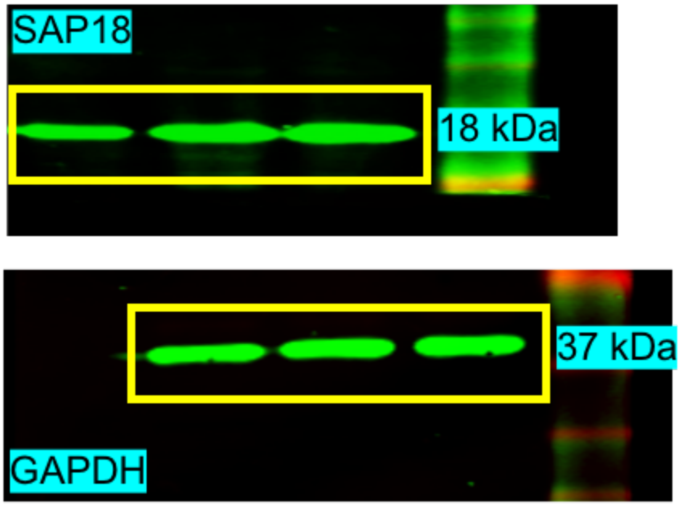

Figure 4a

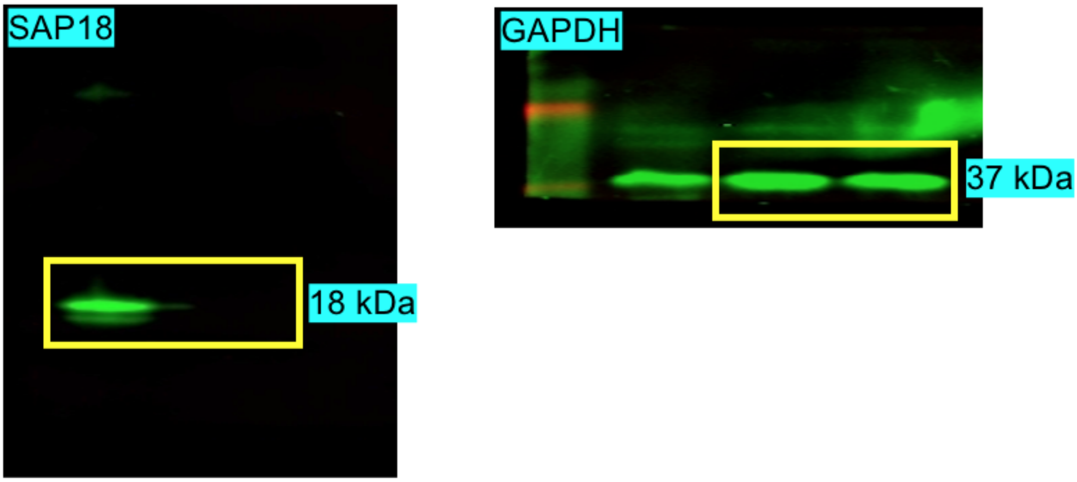

Figure 4g

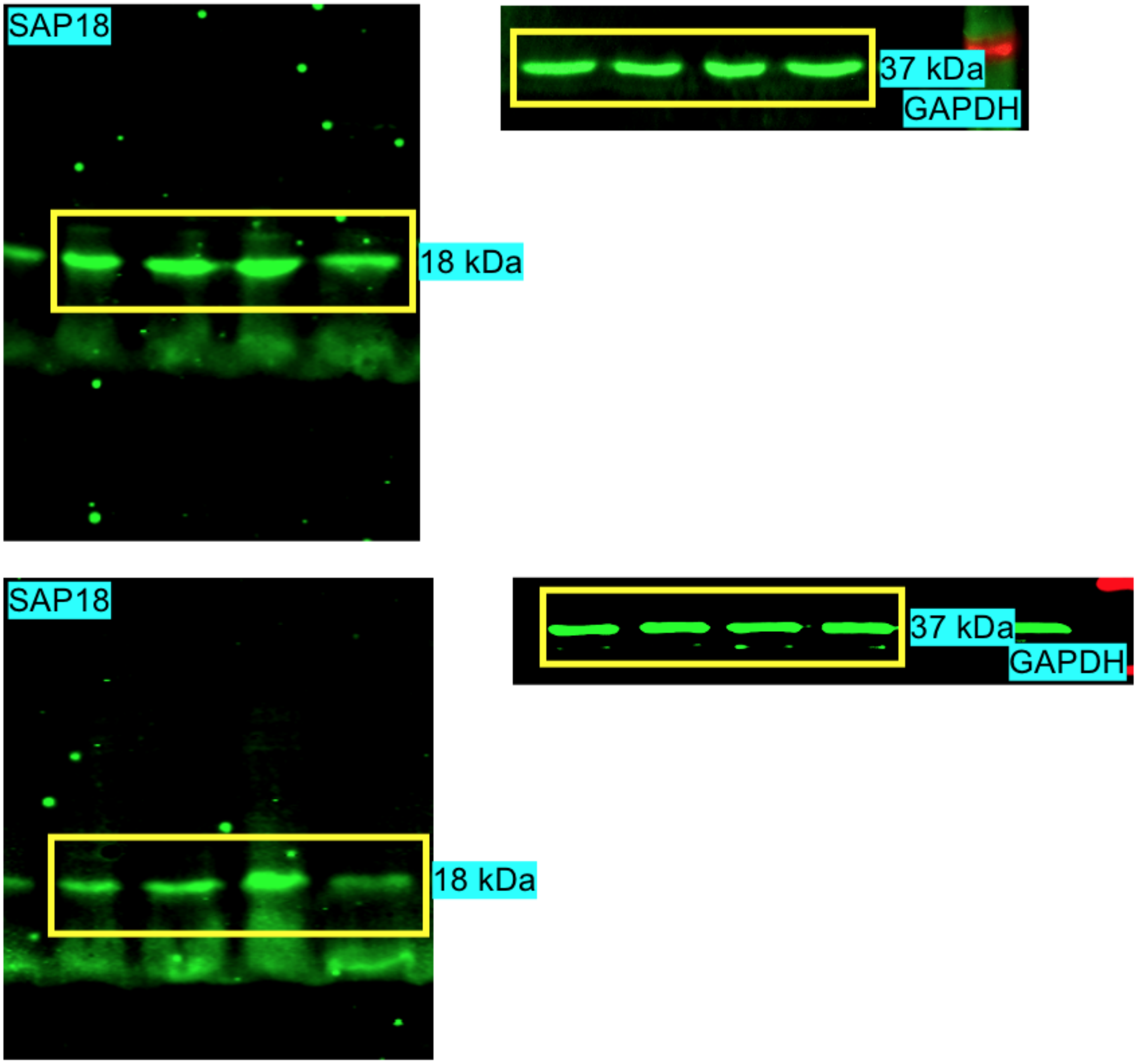

Figure 5a

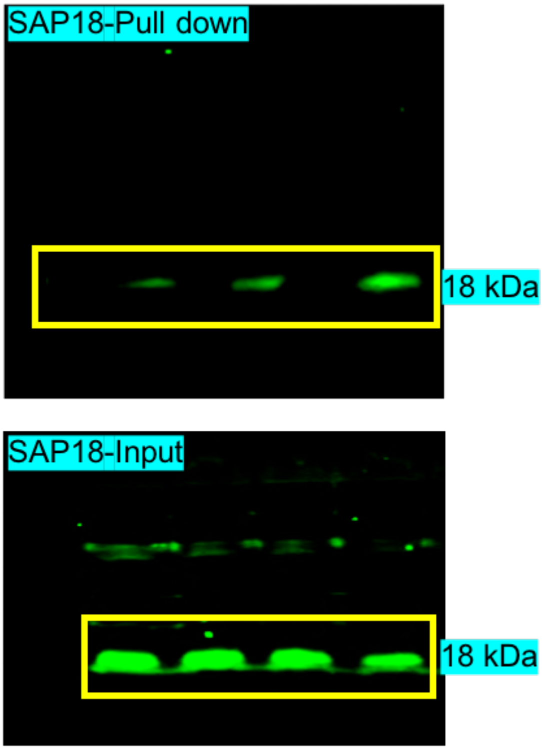

Figure 5b

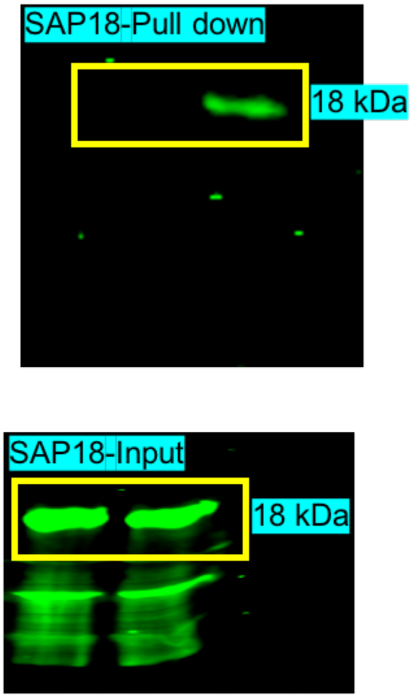

Figure 5c

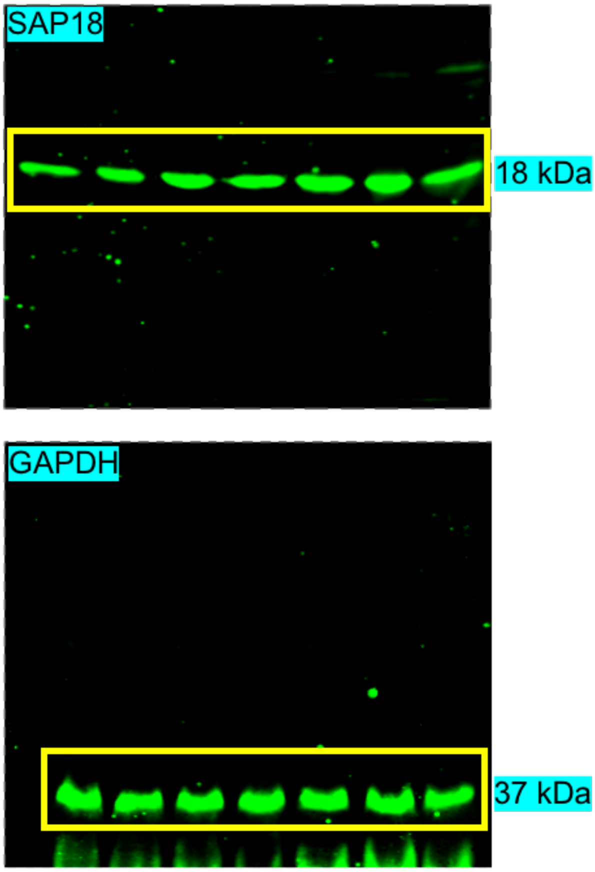

Figure 5h

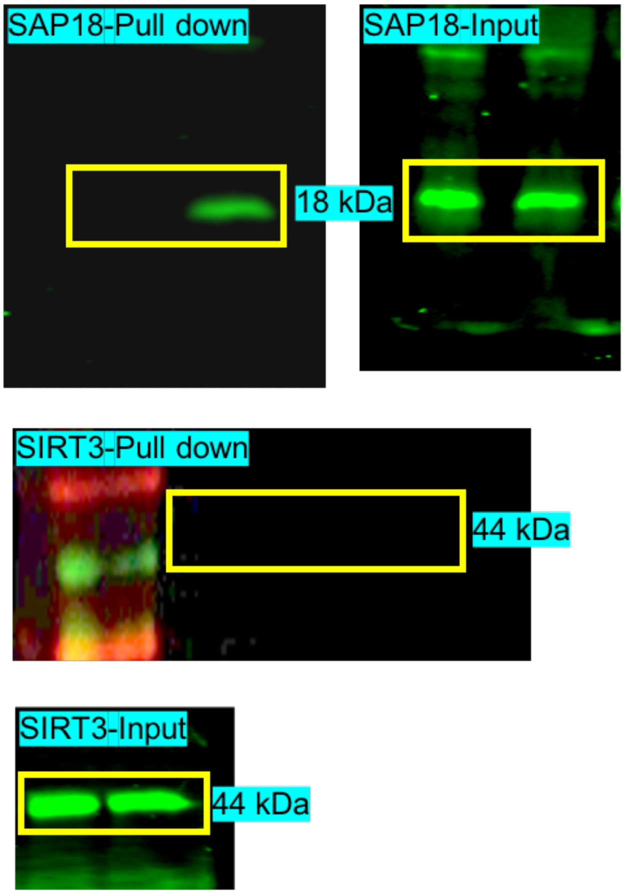

Supplementary Fig. 2e

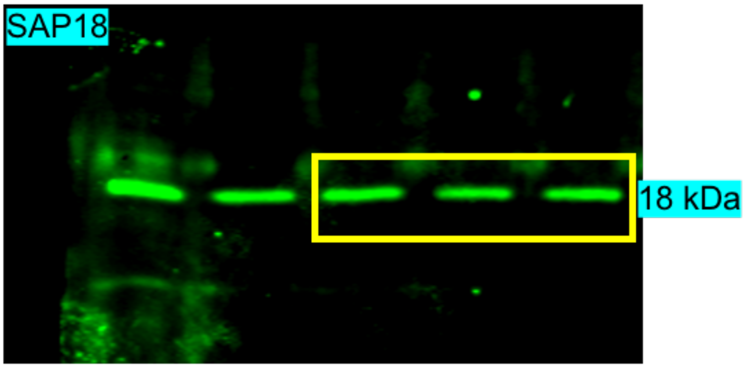

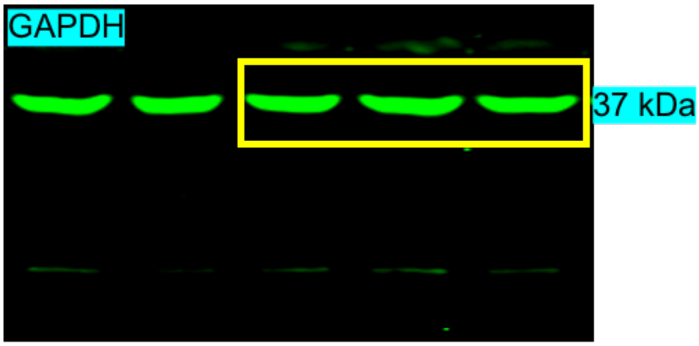

Supplementary Fig. 2f

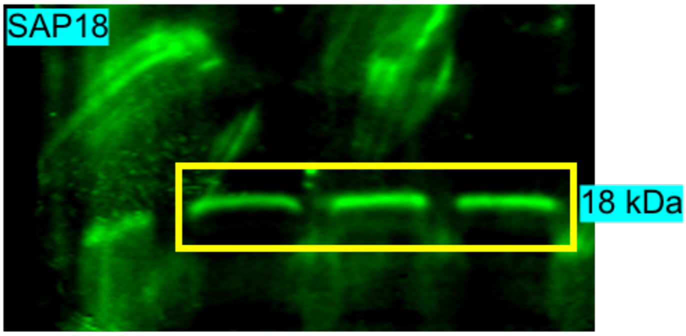

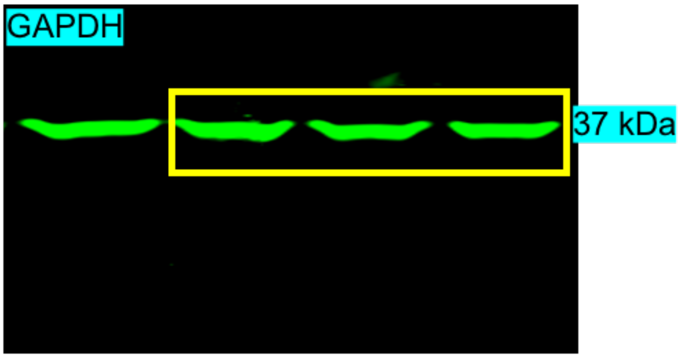

Supplementary Fig. 5a

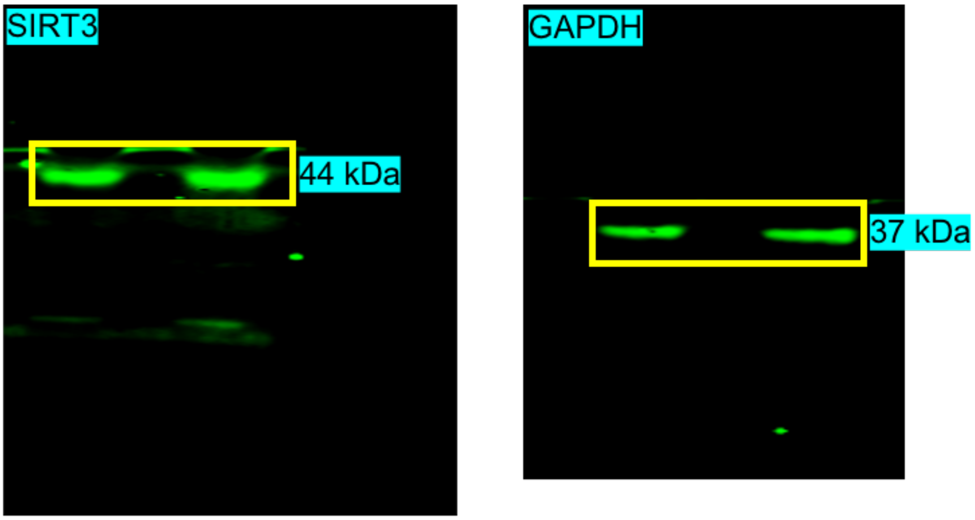

Supplementary Fig. 7a

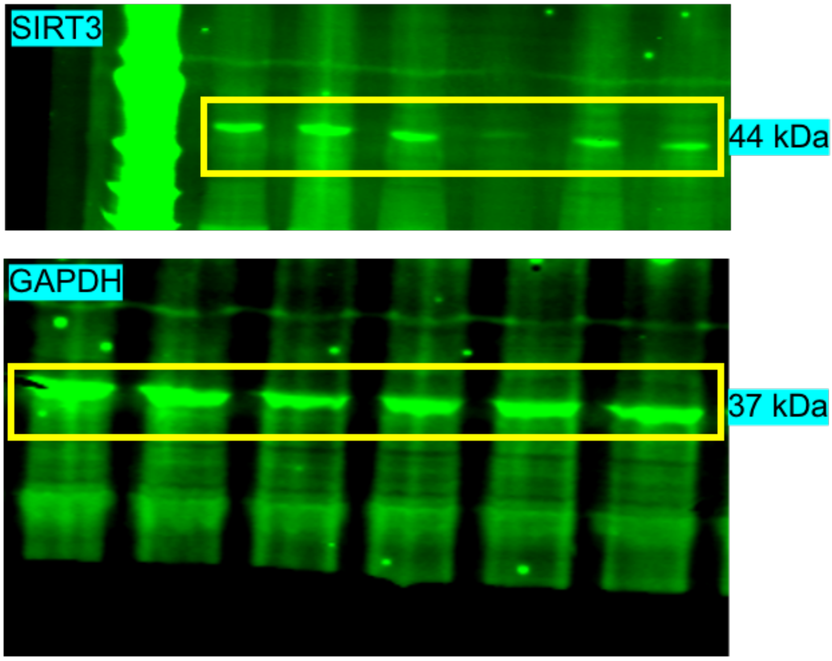

Supplementary Fig. 7b

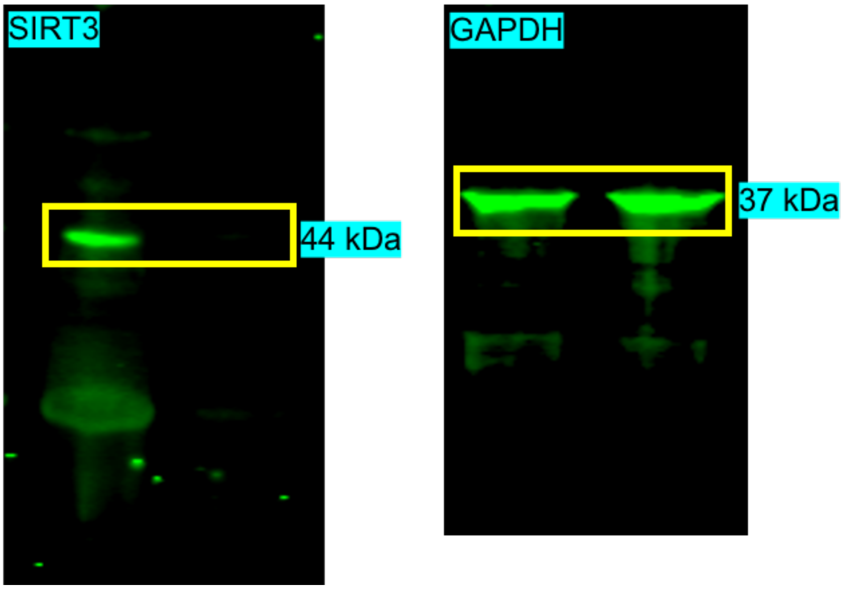

Supplementary Fig. 9

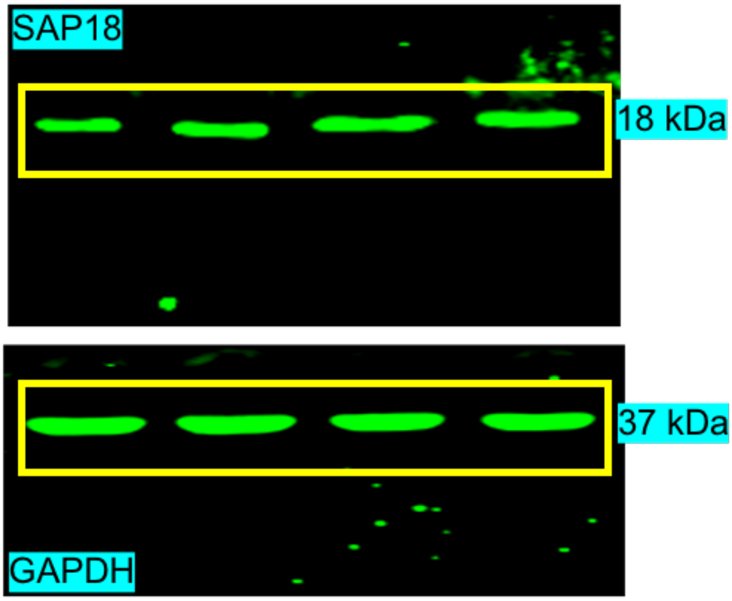

Supplementary Fig. 10a

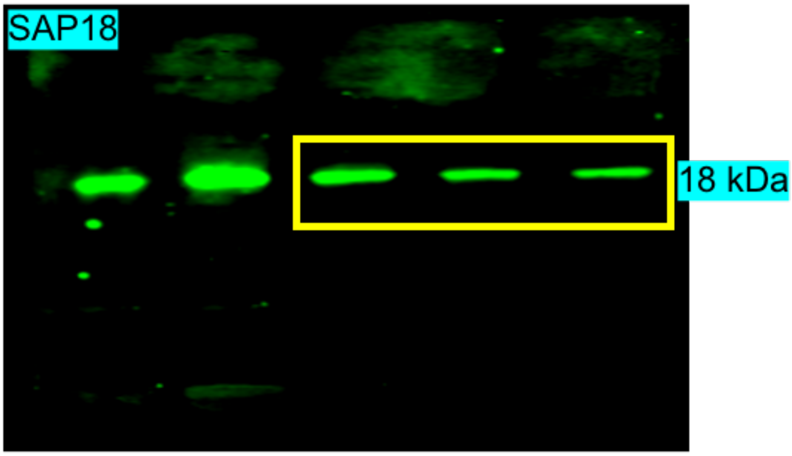

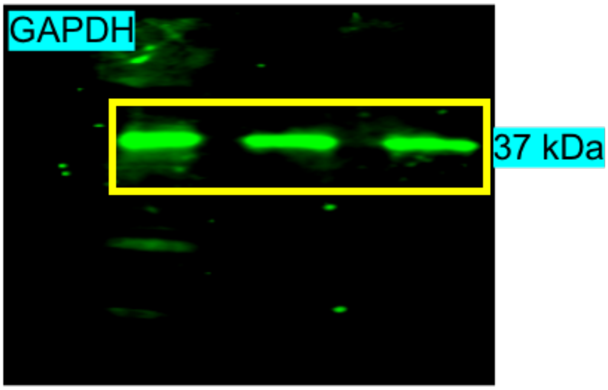

Supplementary Fig. 12

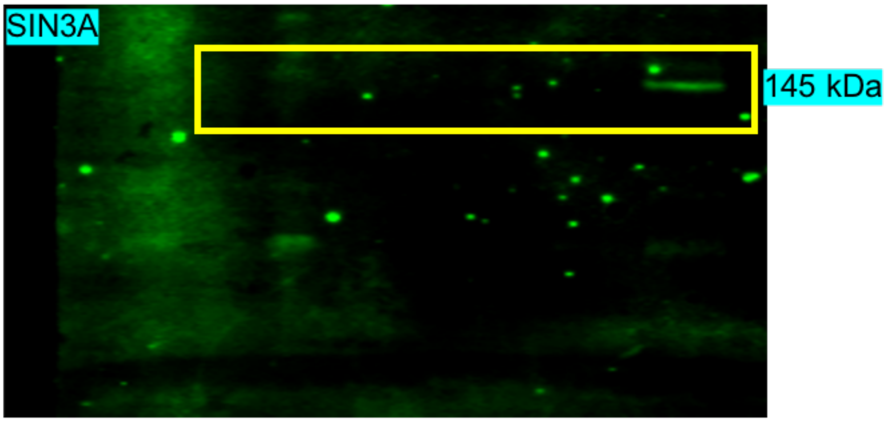

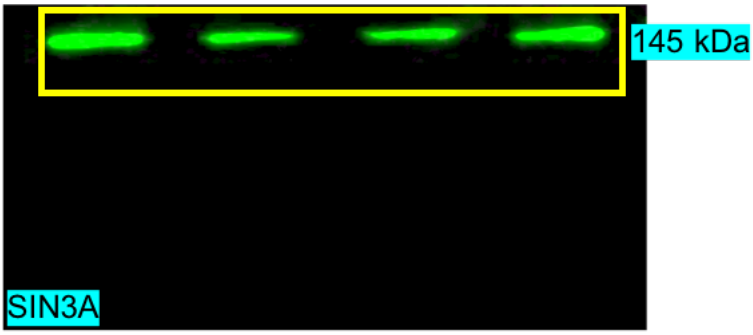

Antibody dilutions

pERK	(1/1000)
ERK	(1/1000)
Bcl-2	(1/1000)
pAKT	(1/1000)
AKT	(1/500)
FLI.1	(1/1000)
cMYC	(1/1000)
SAP18	(1/1000)
SIRT3	(1/500)
SIN3A	(1/1000)
GAPDH	(1/1000)

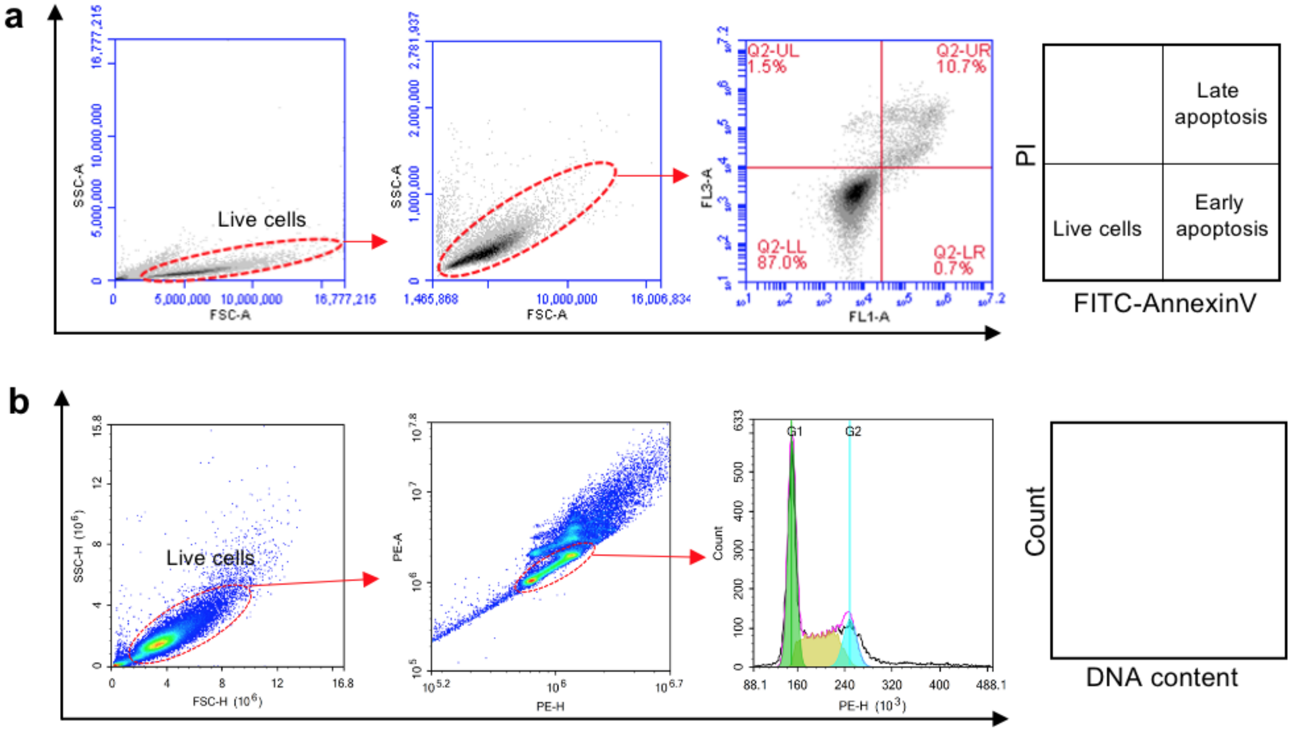

Flow cytometry gating strategy. a, Apoptosis analysis using FITC-AnnexinV/PI. Viable, late and early apoptotic fractions were identified using Annexin V. **b,** Cell cycle analysis using PI. The plot shows cells in G0/G1 phase (green), S phase (yellow) and G2/M phase (blue).

Supplementary Note

Part I Synthesis of new compounds (**A670-A672**)

Part II NMR Spectra and ESI-MS of new compounds

Part III The structure of known compounds

Part I Synthesis of new compounds (**A670-A672**)

1. General

Reagents were purchased from commercial sources (JK chemical, China; Tansoole, China) and used as received. All anhydrous solvents were dried and purified by standard techniques just before use. ^{1}H and ^{13}C NMR spectra were recorded on INOVA-400 MHz NMR spectrometer or Bruker DRX 500 MHz spectrometers with tetramethylsilane (TMS) as the internal standard (Bruker, Bremerhaven, Germany). Chemical shift values (δ) were given in parts per million (ppm). Thin-layer chromatography (TLC) was performed on silica gel GF_{254} (Qingdao Marine Chemical Ltd. P.R. China). Column chromatography (CC) was performed over silica gel (200–300 mesh, Qingdao Marine Chemical Ltd.). ESIMS were recorded on a Finnigan MAT 90 instrument and VG Auto Spec-3000 spectrometer.

2. Synthesis and characterization of A670-A672

Gagamine was isolated from the root of *Cynanchum wilfordii* and had the purity of > 98.0%[1]. The acylation products **A670 and A671**, and the halogenic derivative **A672** were synthesized using a previously reported procedure and the analytical data that we collected were consistent with the reported values[2,3].

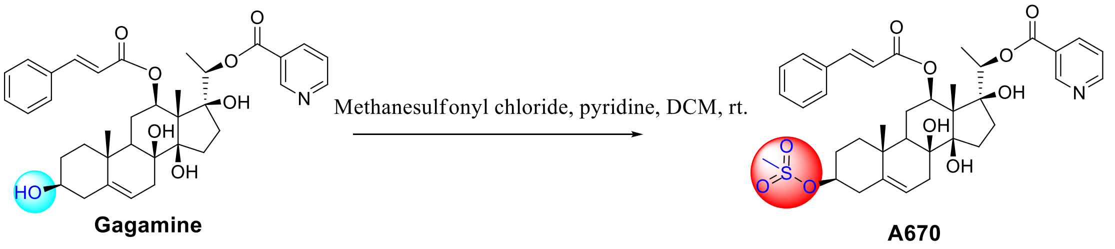

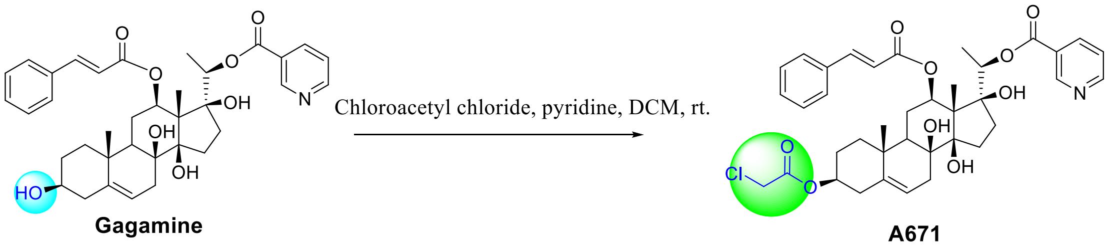

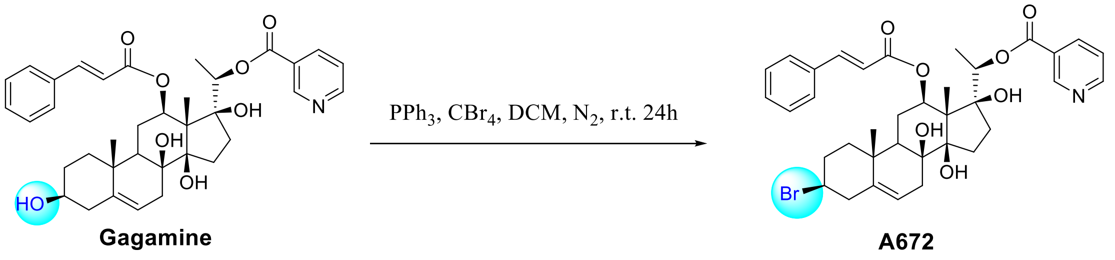

Scheme 1. Synthesis of compound **A670-A672**.

3-O-methanesulfonyl-gagamine (**A670**). To a solution of gagaminine (100 mg, 0.162 mmol) in dry DCM (10 mL), methanesulfonyl chloride (0.5 mL),and pyridine (10 drops) was added. The reaction mixture was stirred for 24 h at room temperature under nitrogen atmosphere. When the starting material was exhausted, the reaction mixture poured into H_2O and extracted with DCM (3 × 30 mL). The organic layer was successively washed with saturated aqueous tartaric acid (3 × 30 mL) and $NaHCO_3$ (3 × 30 mL), and saturated NaCl (2 × 10 mL), respectively. The organic layer was dried over anhydrous $MgSO_4$ and the solvent was removed under reduced pressure. The crude was loaded onto a silica gel column, and eluted with petroleum ether/acetone (80:20) to afford **A670** (60 mg, 53.3 %) as a white amorphous power; ^{1}H NMR (500 MHz, $CDCl_3$) δ 9.19 (s, 1H, H-3″), 8.74-8.73 (d, J = 3.1 Hz, 1H, H-5″), 8.11-8.09 (d, J = 7.95 Hz, 1H, H-7″), 7.41-7.38 (d, J = 16.0 Hz, 1H, H-3′), 7.38-7.33 (m, 3H, Ar-H, H-6″), 7.25-7.19 (m, 3H, Ar-H), 6.11-6.07 (d, J = 15.95 Hz, 1H, H-2′), 5.47 (br.s, 1H, H-6), 4.93-4.89 (q, J = 6.15 Hz, 1H, H-20), 4.85-4.82 (dd, J = 11.25, 4.1 Hz, 1H, H-12), 4.61 (s, H, -OH), 3.88 (brs, H, -OH), 4.57-4.52 (m, 1H, H-3), 3.02 (s, 3H, -$SO_2\underline{CH_3}$), 1.63 (s, 3H, 18-CH_3), 1.38-1.37 (d, J = 6.15 Hz, 3H, 21-CH_3), 1.15 (s, 3H, 19-CH_3); ^{13}C NMR (125 MHz, $CDCl_3$) δ 166.24 (C-1′), 163.51 (C-1″), 153.00 (C-5″), 150.42 (C-3″), 144.33 (C-3′), 137.76 (C-5), 137.61 (C-7″), 133.99 (C-4′), 130.22 (C-7′), 128.77 (C-6′, 8′), 127.97 (C-5′, 9′), 126.27 (C-2″), 123.41 (C-6″), 120.33 (C-2′), 118.61 (C-6), 87.80 (C-14), 87.49 (C-17), 81.33 (C-3), 75.77 (C-20), 74.00 (C-8), 73.26 (C-12), 56.41 (C-13), 43.06 (C-9), 38.99 (C-4), 38.24 (C-1), 36.63 (C-10), 34.36 (C-7), 32.94 (C-16), 32.62 (C-15), 28.12 (C-2), 24.80 (C-11), 18.02 (C-19), 14.96 (C-21), 10.58 (C-18); ESI-MS m/z 696.3 $[M + H]^+$, 718.3 $[M +Na]^+$, 1413.3 $[2M+Na]^+$.

3-O-chloroacetyl-gagamine (**A671**). To a solution of gagaminine (100 mg, 0.162 mmol) in dry DCM (10 mL), methanesulfonyl chloride (0.3 mL),and pyridine (5 drops) was added. The reaction mixture was stirred for 24 h at room temperature under nitrogen atmosphere. When the starting material was exhausted, the reaction mixture poured into H_2O and extracted with DCM (3 × 30 mL). The organic layer was successively washed with saturated aqueous tartaric acid (3 × 30 mL) and $NaHCO_3$ (3 × 30 mL), and saturated NaCl (2 × 10 mL), respectively. The organic layer was dried over anhydrous $MgSO_4$ and the solvent was removed under reduced pressure. The crude was loaded onto a silica gel column, and eluted with petroleum ether/acetone (60:10) to afford **A671** (66 mg, 58.9 %) as a white amorphous power; ^{1}H NMR (500 MHz, $CDCl_3$) δ 9.19 (s, 1H, H-3″), 8.74 (d, J = 3.7 Hz, 1H, H-5″), 8.11-8.09 (t, J = 7.8 Hz, 1H, H-7″), 7.41-7.38 (d, J = 16.4 Hz, 1H, H-3′), 7.36-7.32 (m, 3H, Ar-H), 7.25-7.24 (m, 2H, Ar-H), 7.21-7.19 (dd, J = 7.8, 4.9 Hz, 1H, H-6″), 6.10-6.07 (d, J = 16.0 Hz, 1H, H-2′), 5.44 (br.s, 1H, H-6), 4.93-4.89 (q, J = 5.95 Hz, 2H, H-20), 4.85-4.82 (dd, J =11.25, 4.1 Hz, 1H, H-12), 4.76-4.69 (m, 1H, H-3), 4.55 (s, H, -OH), 4.04 (s, 2H, -CO$\underline{CH_2}$Cl), 3.90 (s, H, -OH), 1.64 (s, 3H, 18-CH_3), 1.38-1.37 (d, J = 6.15 Hz, 3H, 21-CH_3), 1.16 (s, 3H, 19-CH_3); ^{13}C NMR (100 MHz, $CDCl_3$) δ 166.7 (-$\underline{C}$OCH_2Cl), 166.3 (C-1′), 163.6 (C-1″), 153.3 (C-5″), 150.6 (C-3″), 144.3 (C-3′), 138.4 (C-5), 137.4 (C-7″), 134.0 (C-4′), 130.3 (C-7′), 128.7 (C-6′, 8′), 128.0 (C-5′, 9′), 126.1 (C-2″), 123.3 (C-6″), 119.6 (C-6), 118.6 (C-2′), 87.8 (C-14), 87.4 (C-17), 75.8 (C-20), 75.7 (C-3), 74.1 (C-8), 73.3 (C-12), 56.4 (C-13), 43.1 (C-9), 41.1 (C-4), 38.2 (C-1), 37.6 (-CO$\underline{CH_2}$Cl), 36.8 (C-10), 34.3 (C-7), 32.8 (C-16), 32.7 (C-15), 26.9 (C-2), 24.8 (C-11), 18.1 (C-19), 14.9 (C-21), 10.5 (C-18); ESI-MS *m/z* 694.2 $[M + H]^+$, 716.0 $[M + Na]^+$, 1409.3 $[2M + Na]^+$.

at room temperature in DCM,

3-Bromidegagamine (**A672**). To a solution of gagaminine (100 mg, 0.162 mmol) in dry DCM (10 mL), triphenylphosphine (PPh_3, 90 mg, 0.340 mmol), carbontetrabromide (CBr_4, 161 mg, 0.486 mmol) was added. The reaction mixture was stirred for 24 h at room temperature under nitrogen atmosphere. When the starting material was exhausted, the reaction mixture poured into H_2O and extracted

with DCM (3 × 30 mL). The organic layer was successively washed with saturated aqueous tartaric acid (3 × 30 mL) and $NaHCO_3$ (3 × 30 mL), and saturated NaCl (2 × 10 mL), respectively. The organic layer was dried over anhydrous $MgSO_4$ and the solvent was removed under reduced pressure. The crude was loaded onto a silica gel column, and eluted with petroleum ether/acetone (90:10) to afford **A672** (95 mg, 86.4 %) as a white amorphous power; ^{1}H NMR(500 MHz, $CDCl_3$) δ 9.19 (s, 1H, H-3″), 8.73-8.72 (d, *J* = 3.65 Hz, 1H, H-5″), 8.11-8.09 (tt, *J* = 7.85, 1.8, 1.8 Hz, 1H, H-4″), 7.41-7.37 (d, *J* = 15.95 Hz, 1H, H-3′), 7.39-7.32 (m, 3H, Ar-H, H-6″), 7.25-7.18 (m, 3H, Ar-H), 6.10-6.07 (d, *J* = 15.9 Hz, 1H, H-2′), 5.40 (br.s, 1H, H-6), 4.92-4.89 (q, *J* = 6.05 Hz, 1H, H-20), 4.84-4.81 (dd, *J* = 11.5, 4.45 Hz, 1H, H-12), 4.59 (s, H, -OH),3.96 (brs, H, -OH), 3.94-3.89 (m, 1H, H-3),1.63 (s, 3H, 18-CH_3),1.38-1.37 (d, *J* = 6.05 Hz, 3H, 21-CH_3), 1.17 (s, 3H, 19-CH_3); ^{13}C NMR (125 MHz, $CDCl_3$) δ 166.26 (C-1′), 163.64 (C-1″), 153.15 (C-5″), 150.59 (C-3″), 144.30 (C-3′), 140.76 (C-5), 137.42 (C-7″), 134.00 (C-4′), 130.28 (C-7′), 128.74 (C-6′, 8′), 127.97 (C-5′, 9′), 126.21 (C-2″), 123.31 (C-6″), 118.79 (C-2′), 118.63 (C-6), 87.86 (C-14), 87.41 (C-17), 75.72 (C-20), 73.96 (C-8), 73.31 (C-12), 56.43 (C-13), 51.63 (C-3), 43.93 (C-9), 43.31 (C-4), 41.72 (C-1), 36.67 (C-10), 34.24 (C-7), 33.42 (C-16), 32.85 (C-15), 32.74 (C-2), 24.72 (C-11), 18.13 (C-19), 14.95 (C-21), 10.53 (C-18); ESI-MS *m/z* 682.2 $[M + 2H]^+$, 702.2 $[M+Na]^+$, 1381.2 $[2M+Na]^+$.

Part II NMR Spectra and ESI-MS of new compounds

500 MHz ¹H NMR Spectrum of ***3-O-methanesulfonyl-gagamine*** (**A670**) in $CDCl_3$

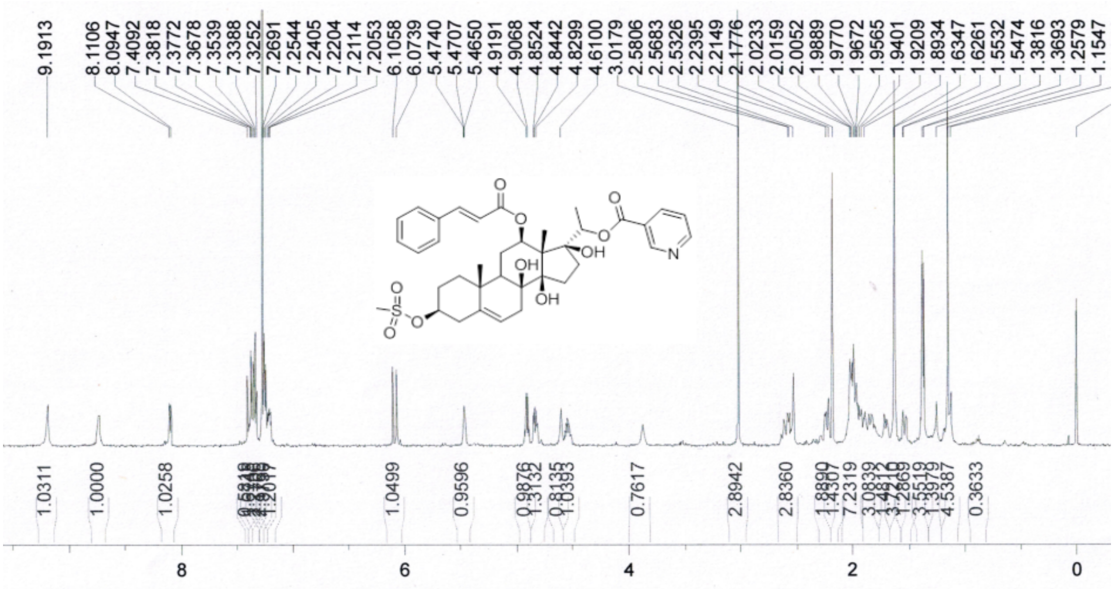

125 MHz ¹³C NMR Spectrum of ***3-O-methanesulfonyl-gagamine*** (**A670**) in $CDCl_3$

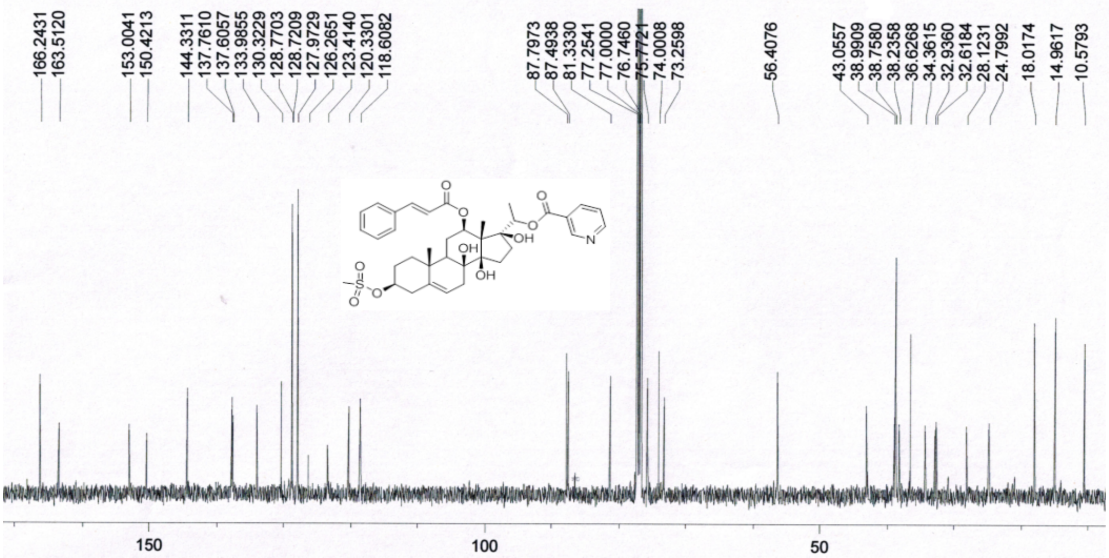

ESI-MS of ***3-O-methanesulfonyl-gagamine*** (**A670**)

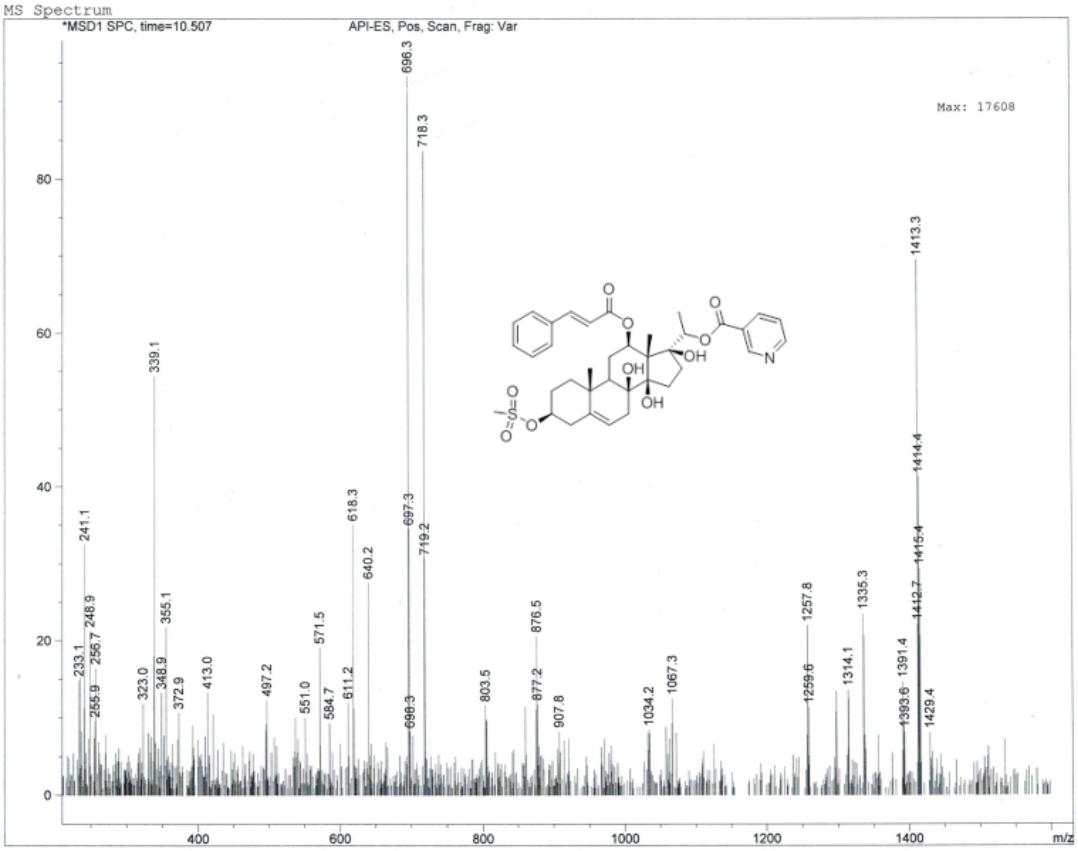

500 MHz ^{1}H NMR Spectrum of ***3-O-chloroacetyl-gagamine*** (**A671**) in $CDCl_3$

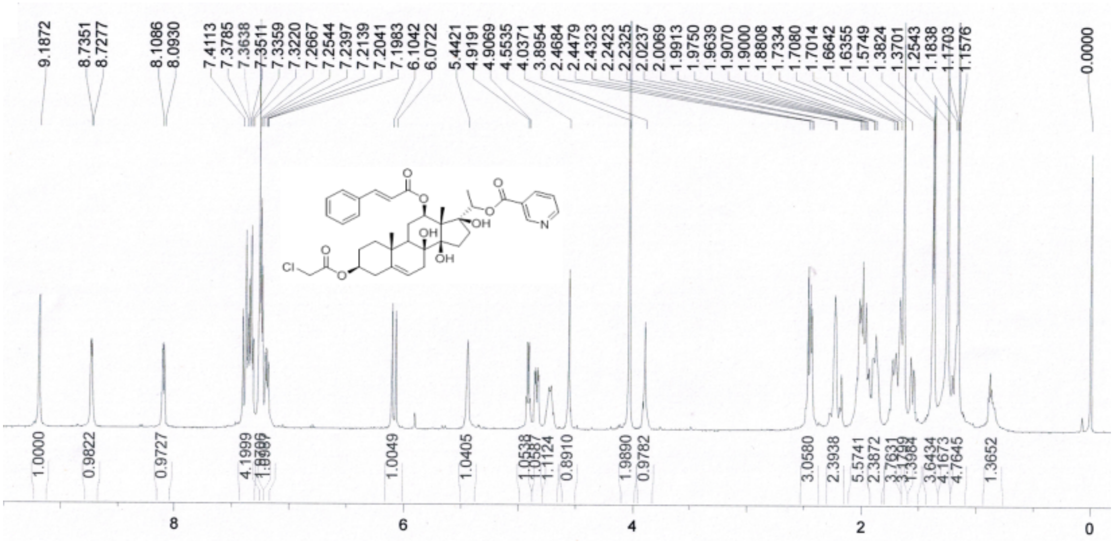

100 MHz ^{13}C NMR Spectrum of ***3-O-chloroacetyl-gagamine*** (**A671**) in $CDCl_3$

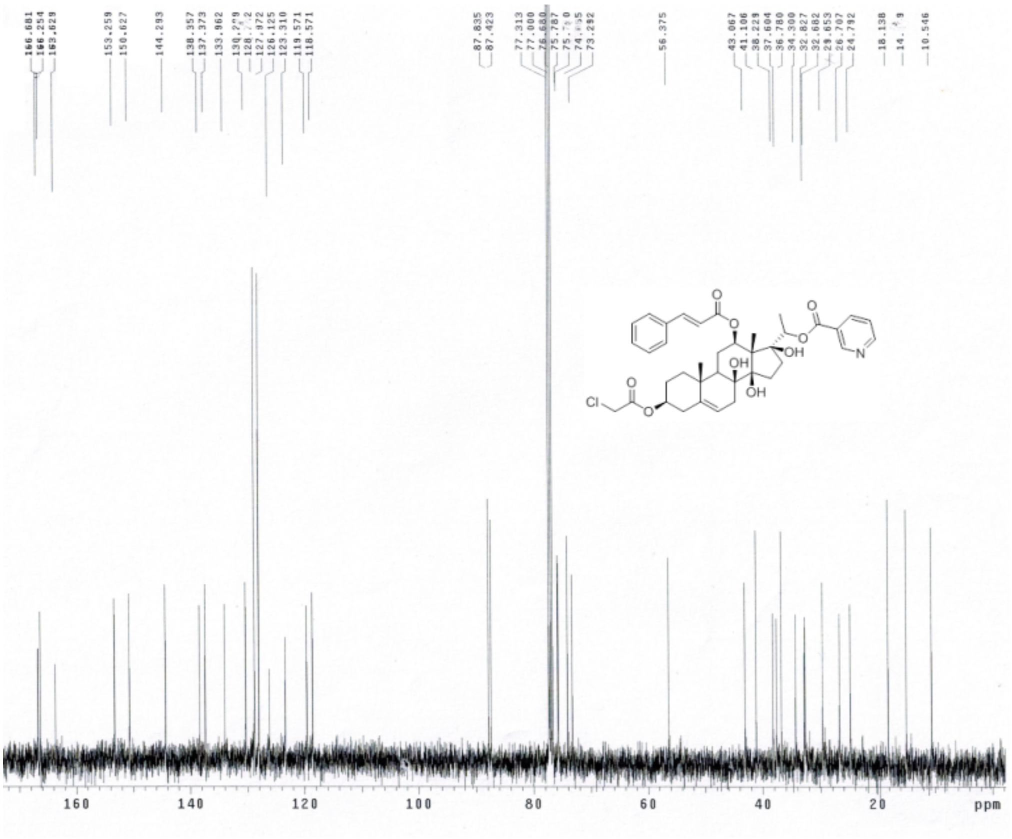

ESI-MS of ***3-O-chloroacetyl-gagamine*** (**A671**)

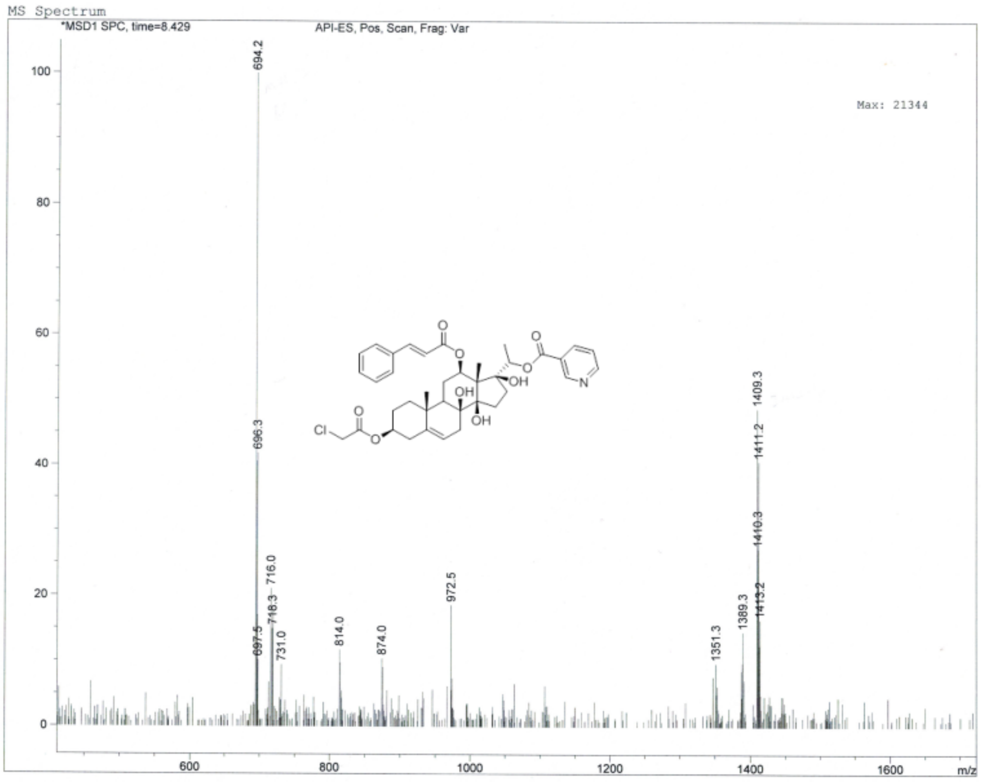

500 MHz ^{1}H NMR Spectrum of ***3-Bromidegagamine*** (**A672**) in $CDCl_3$

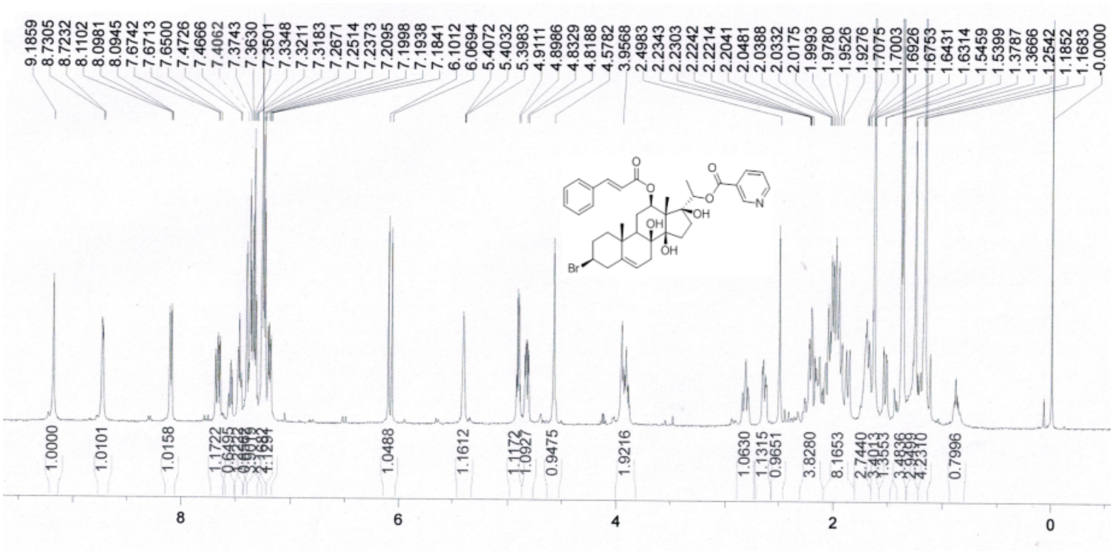

125 MHz ^{13}C NMR Spectrum of ***3-Bromidegagamine*** (**A672**) in $CDCl_3$

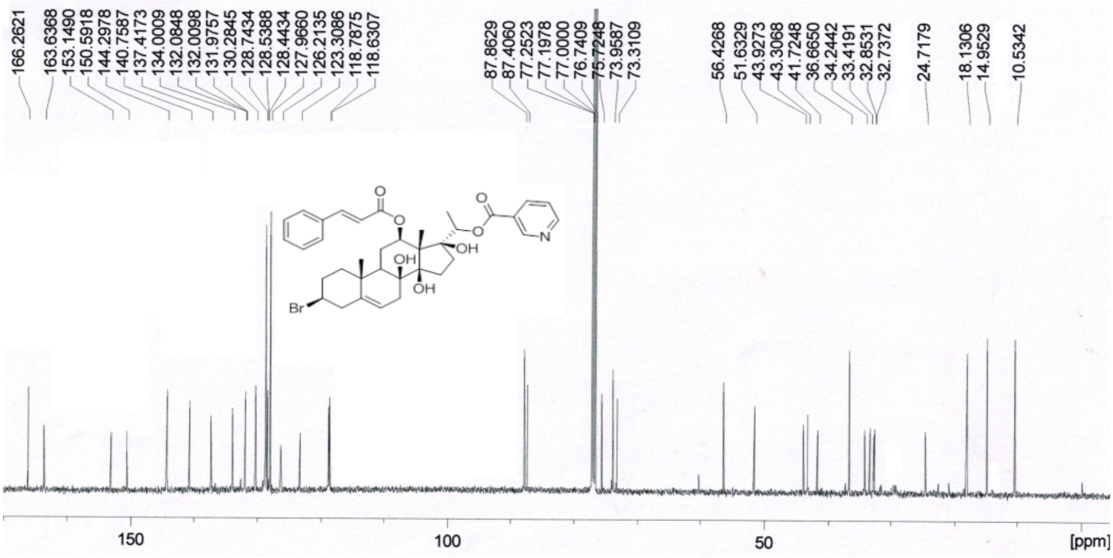

ESI-MS of ***3-Bromidegagamine*** (**A672**)

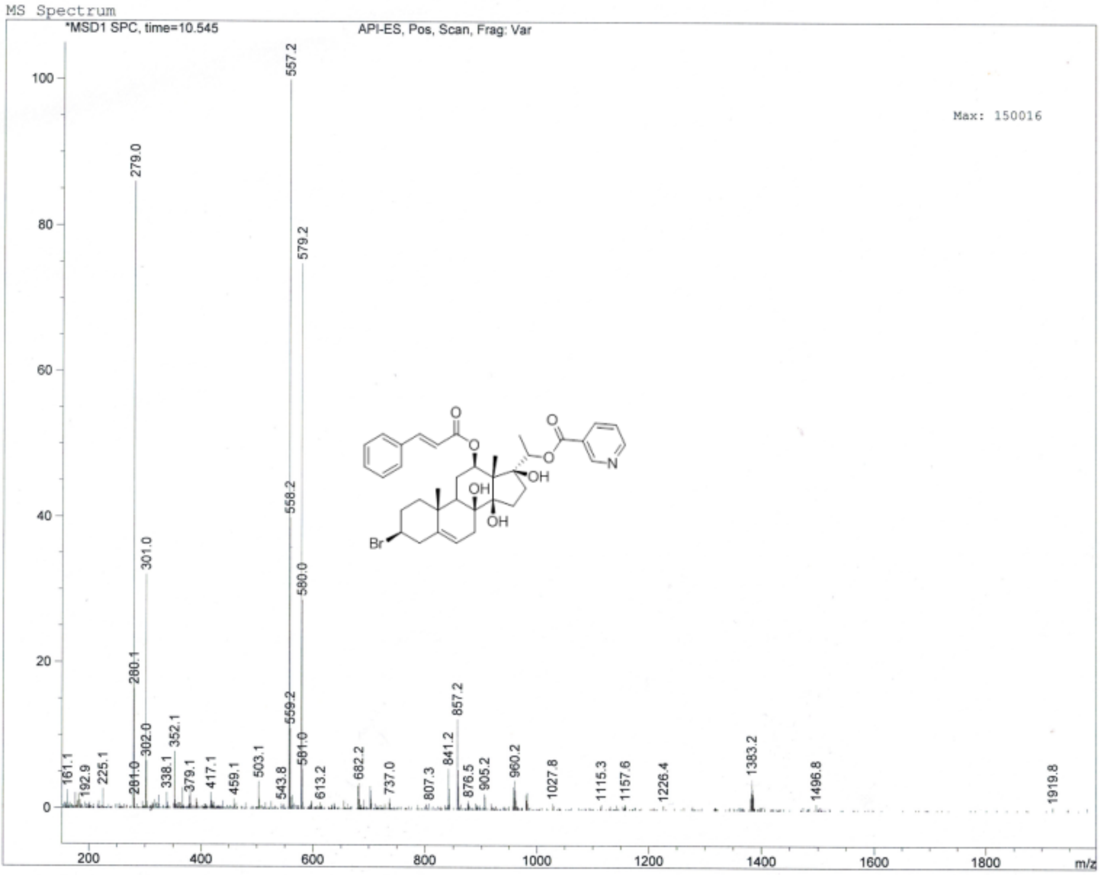

Part III The structure of known compounds **(A601-A669)**

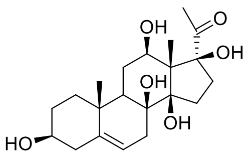
A601

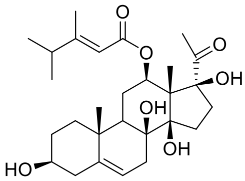
A602

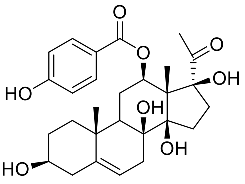
A603

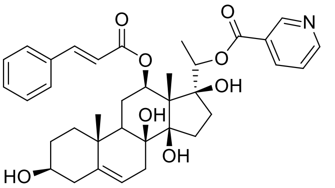
A604

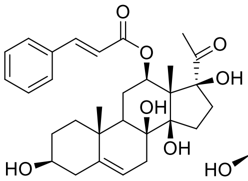
A605

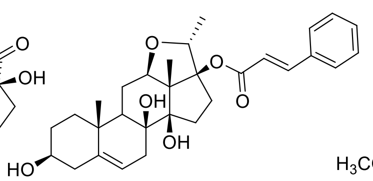
A606

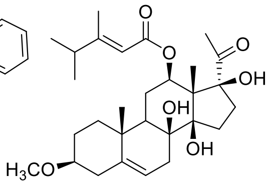
A607

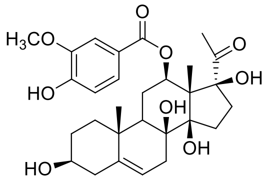
A608

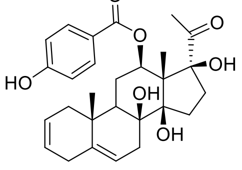
A609

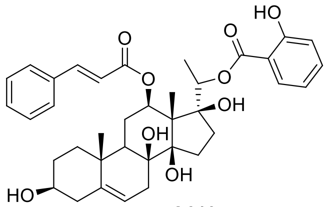
A610

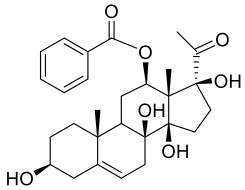
A611

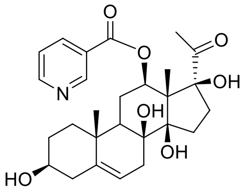
A612

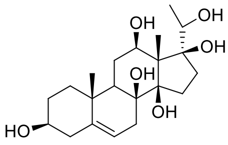
A613

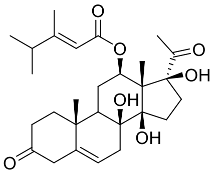
A614

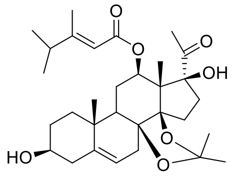
A615

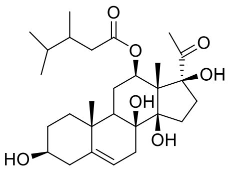
A616

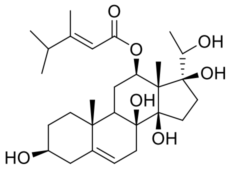
A617

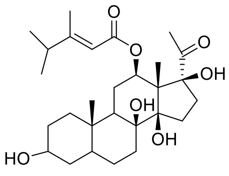
A618

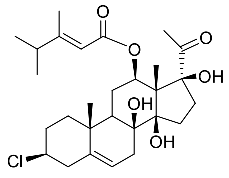
A619

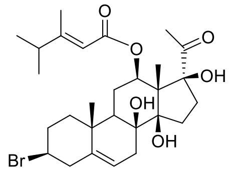
A620

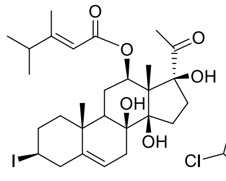
A621

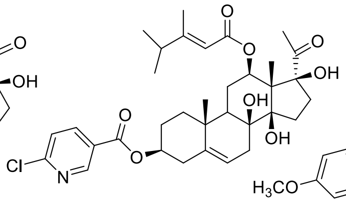
A622

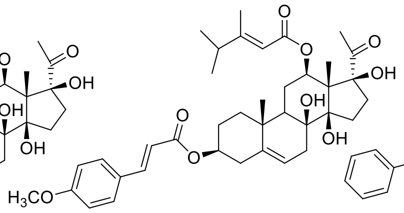
A623

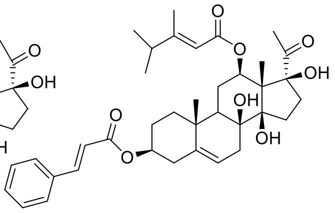
A624

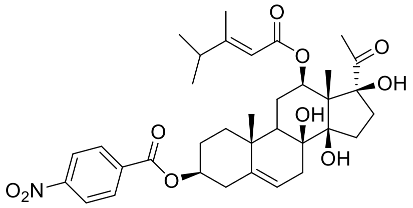
A625

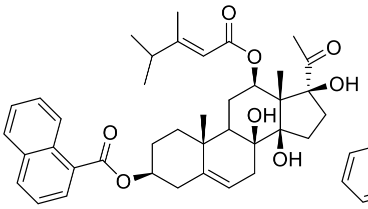
A626

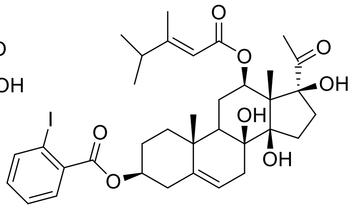
A627

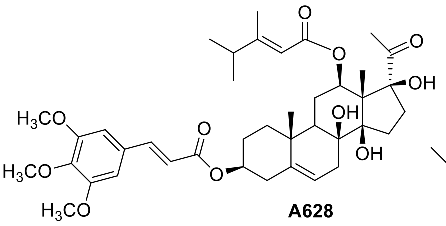

A628

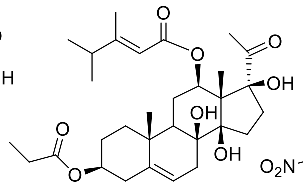

A629

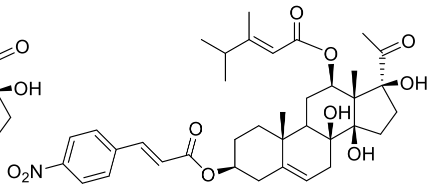

A630

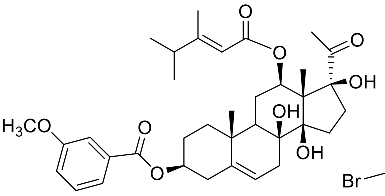

A631

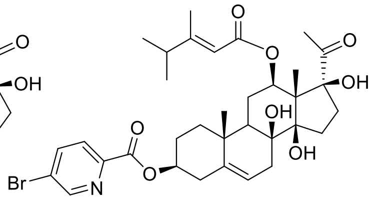

A632

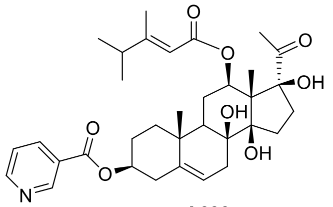

A633

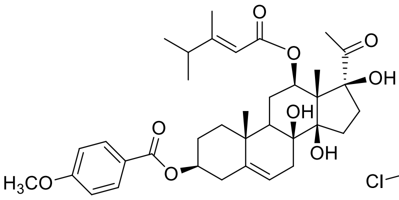

A634

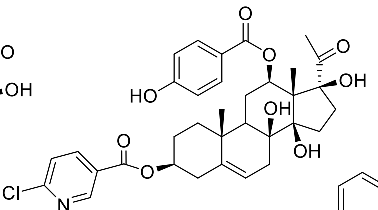

A635

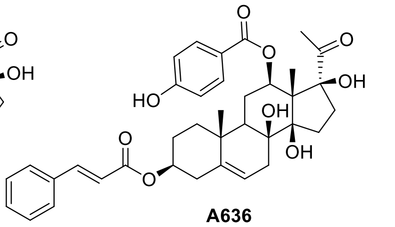

A636

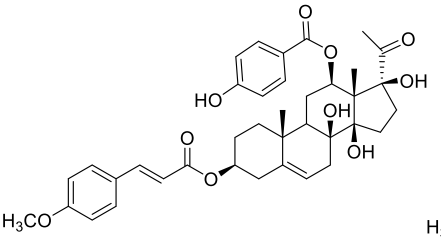

A637

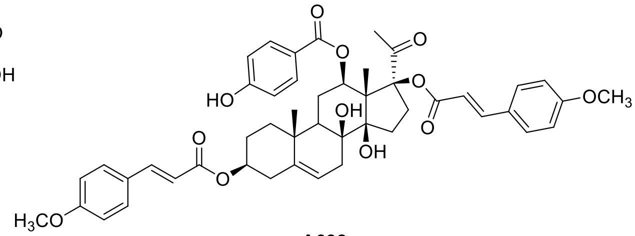

A638

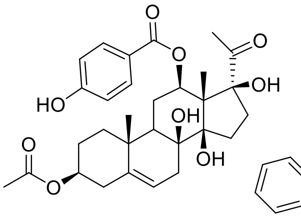

A639

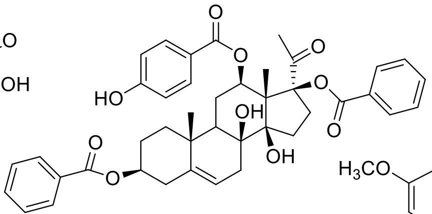

A640

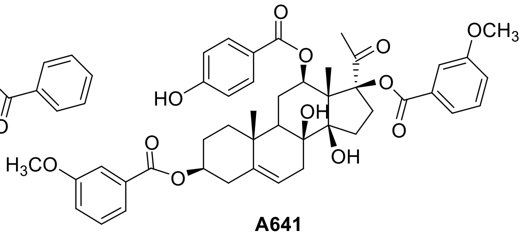

A641

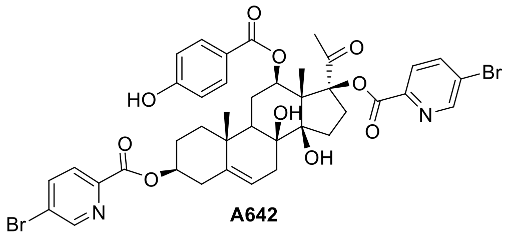

A642

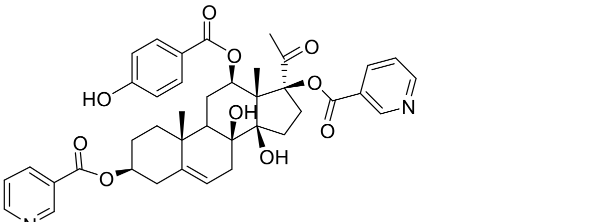

A643

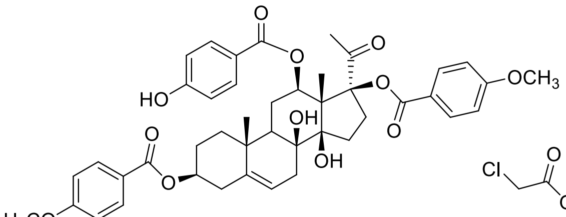

A644

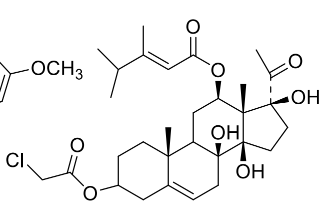

A645

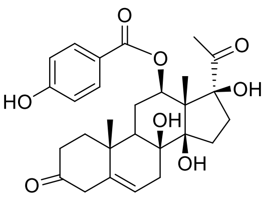

A646

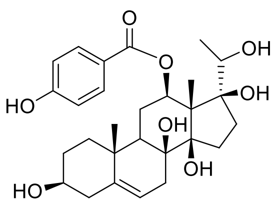

A647

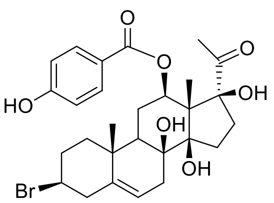

A648

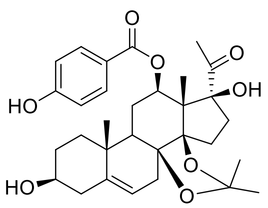

A649

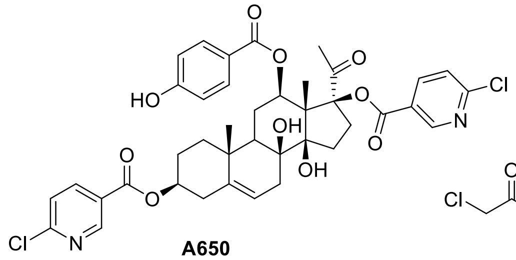

A650

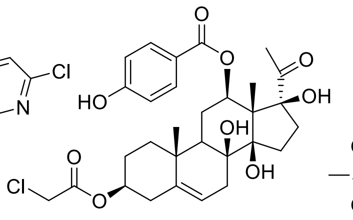

A651

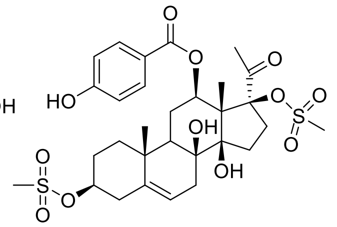

A652

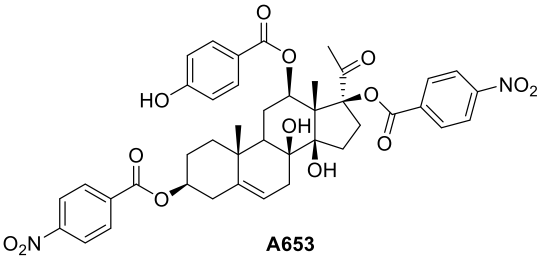

A653

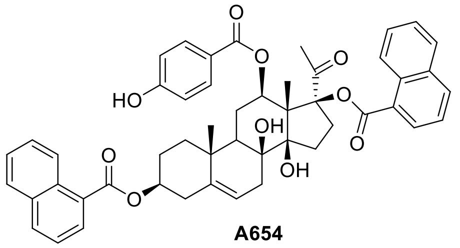

A654

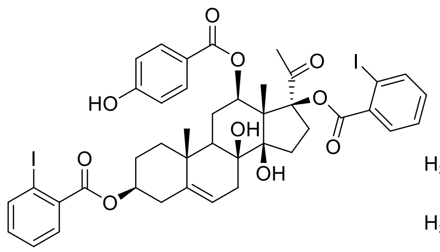

A655

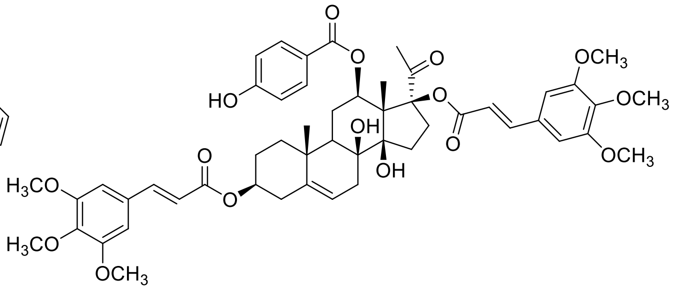

A656

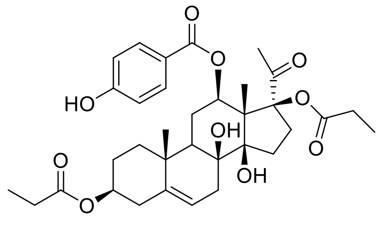

A657

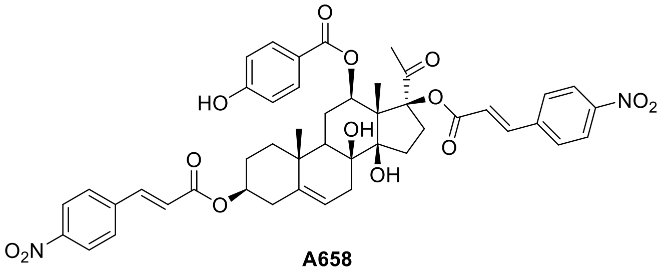

A658

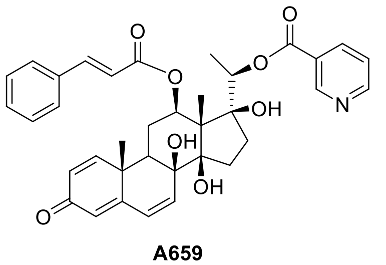

A659

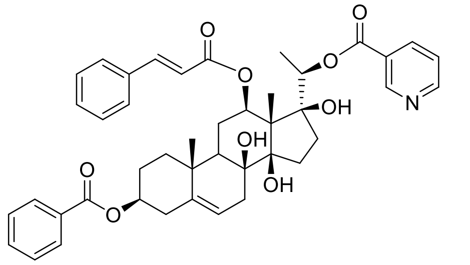

A660

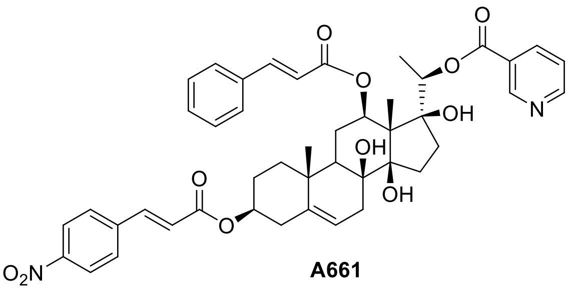

A661

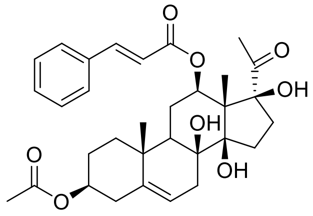

A662

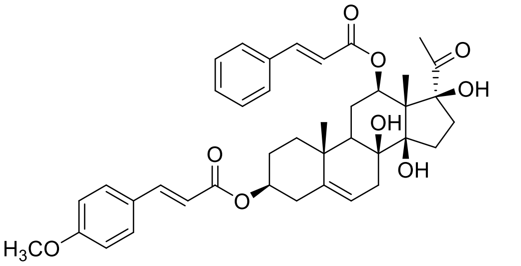

A663

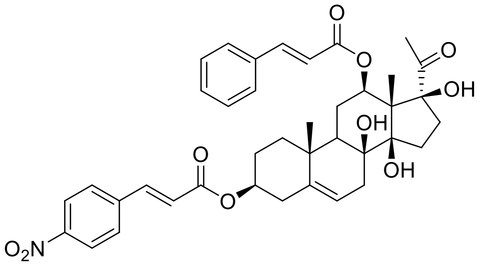

A664

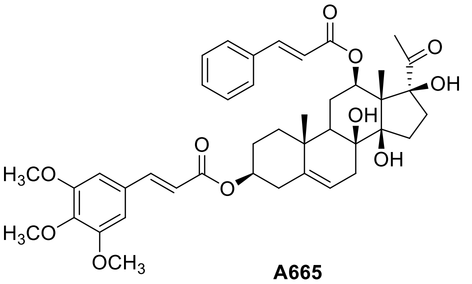

A665

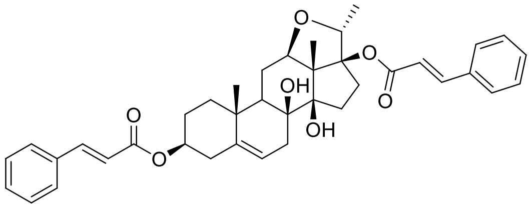

A666

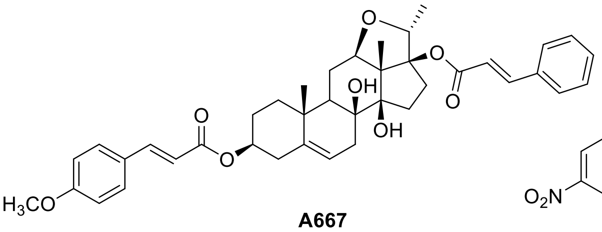

A667

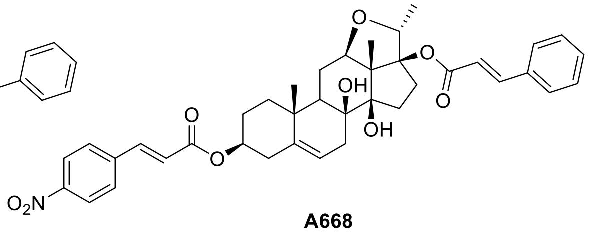

A668

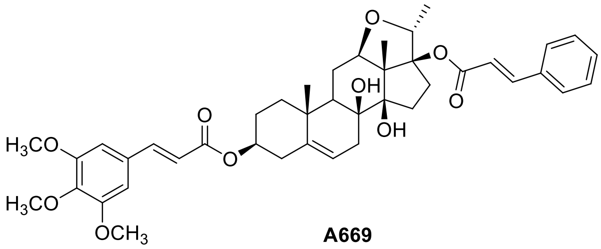

A669

Figure 1. Known C_{21}-steroidal derivatives. [1-3]

References

[1] Huang, L.J. et al. Studies on cytotoxic pregnane sapogenins from *Cynanchum wilfordii.* Fitoterapia **101,** 107-116 (2015).

[2] Huang, L.J. et al. Synthesis and evaluation of antifungal activity of C_{21}-steroidal derivatives. Bioorganic and Medicinal Chemistry Letters **26,** 2040-2043 (2016).

[3] Huang, L.J. et al. C_{21}-steroidal pregnane sapogenins and their derivatives as anti-inflammatory agents. Bioorganic and Medicinal Chemistry **25(13),** 3512-3524 (2017).
