# Supplementary material for: A C21-steroidal derivative suppresses T-cell lymphoma in mice by inhibiting SIRT3 via SAP18-SIN3
Source: Commun Biol. 2020 Dec 3;3:732. doi: 10.1038/s42003-020-01458-3 (PMC7713351; doi:10.1038/s42003-020-01458-3)
Supplement: Supplementary file 4 — Description of Additional Supplementary Files [file 42003_2020_1458_MOESM4_ESM.docx]

Description of additional supplementary files

Supplementary Data 1: Source data underlying the graphs presented in the figures.
